# sEMG-based prediction of human forearm movements utilizing a biomechanical model based on individual anatomical/ physiological measures and a reduced set of optimization parameters

**DOI:** 10.1371/journal.pone.0289549

**Published:** 2023-08-03

**Authors:** Nils Grimmelsmann, Malte Mechtenberg, Wolfram Schenck, Hanno Gerd Meyer, Axel Schneider

**Affiliations:** 1 Biomechatronics and Embedded Systems Group, Bielefeld University of Applied Sciences, Bielefeld, NRW, Germany; 2 Institute of Mechatronics and Systems Dynamics, Bielefeld University of Applied Sciences, Bielefeld, NRW, Germany; Università degli Studi di Milano: Universita degli Studi di Milano, ITALY

## Abstract

For assistive devices such as active orthoses, exoskeletons or other close-to-body robotic-systems, the immediate prediction of biological limb movements based on biosignals in the respective control system can be used to enable intuitive operation also by untrained users e.g. in healthcare, rehabilitation or industrial scenarios. Surface electromyography (sEMG) signals from the muscles that drive the limbs can be measured before the actual movement occurs and, hence, can be used as source for predicting limb movements. The aim of this work was to create a model that can be adapted to a new user or movement scenario with little measurement and computing effort. Therefore, a biomechanical model is presented that predicts limb movements of the human forearm based on easy to measure sEMG signals of the main muscles involved in forearm actuation (*lateral* and *long head* of *triceps* and *short* and *long head* of *biceps*). The model has 42 internal parameters of which 37 were attributed to 8 individually measured physiological measures (location of *acromion* at the shoulder, *medial/lateral epicondyles* as well as *olecranon* at the elbow, and *styloid processes* of *radius/ulna* at the wrist; maximum muscle forces of *biceps* and *triceps*). The remaining 5 parameters are adapted to specific movement conditions in an optimization process. The model was tested in an experimental study with 31 subjects in which the prediction quality of the model was assessed. The quality of the movement prediction was evaluated by using the normalized mean absolute error (nMAE) for two arm postures (lower, upper), two load conditions (2 kg, 4 kg) and two movement velocities (slow, fast). For the resulting 8 experimental combinations the nMAE varied between nMAE = 0.16 and nMAE = 0.21 (lower numbers better). An additional quality score (QS) was introduced that allows direct comparison between different movements. This score ranged from QS = 0.25 to QS = 0.40 (higher numbers better) for the experimental combinations. The above formulated aim was achieved with good prediction quality by using only 8 individual measurements (easy to collect body dimensions) and the subsequent optimization of only 5 parameters. At the same time, just easily accessible sEMG measurement locations are used to enable simple integration, e.g. in exoskeletons. This biomechanical model does not compete with models that measure all sEMG signals of the muscle heads involved in order to achieve the highest possible prediction quality.

## 1 Introduction

The model based prediction of limb movements from myoelectric signals as measured in surface electromyography (sEMG) can be used for intuitive human-machine interfaces (HMIs) in close-to-body robotics like in wearables and in actuated orthoses [1–6]. Between the appearance of myoelectric signals (sEMG) and the respective muscle contraction (force generation) as well as between muscle contraction and the actual limb movement, time delays occur. The former delay results from the *activation dynamics* during static or dynamic contractions of the respective muscle which can be explained by the electrochemical conversion of myoelectric excitation into the release of Ca^2+^ ions within the muscle sarcomeres followed by sarcomere shortening (concentric contraction) or sarcomere lengthening (excentric contraction) [7, 8]. The latter delay results from the fact that the inherently inert mechanical system of a limb must be accelerated by muscular forces before motion becomes noticeable in terms of a measurable angular velocity of the respective joint or an interaction force e.g. under isometric conditions. The resulting overall time delays lie in the range of e.g. ∼ 106 ms for the human knee extensor [9] and e.g. ∼ 53 ms for muscles of the upper limb [10–12]. The time interval between innervation and movement can potentially be used for a model-based prediction of the limb movement by using sEMG data as input. In order to exploit this time advantage in the control of close-to-body robotic systems, fast model algorithms with sufficient quality for limb motion prediction are needed to reduce the delay between limb and robot motion. Biomechanical limb models at different levels of detail are available as a basis for the above strategy. These contain Hill-type muscle models [7, 13] as subsystems and cover, e.g. for human upper limbs, aspects like joint geometry and its effect on the muscle moment arm [14], the structure of the musculoskeletal system [15], or, length partitioning and properties of muscle and tendon [16]—the latter also with respect to age [17]. As a consequence, each submodel contributes to a growing range of parameters. These parameters must be individually adjusted to the respective user and, if necessary, continuously readjusted during prolonged use of the model, e.g. due to muscle fatigue [18, 19] or due to electrode shifts on the skin and other interface effects [20, 21].

This work proposes a prediction model for limb movements of the forearm, which is based on sEMG measurements of the heads of the main flexor and extensor muscles (*biceps* and *triceps brachii*). Particular attention was paid to reducing the parameter space as much as possible I) by reducing the number of parameter using prior knowledge—like the time constants in the activation dynamics, and II) by obtaining hard-to-access physiological parameters from easy-to-access physiological/anatomical measures—like locations and directions of palpable bone processes and the respective intermediate distances and rotations (e.g. *acromion* of the *scapula*, *epicondyles* of the *distal humerus* and *styloid process* of *ulna*)—and to adjust the remaining, free parameters to individuals in an optimization process.

Such a model, in which only a few parameters are determined by an optimization process, should be particularly suitable for use in motion prediction scenarios outlined above. The model does not compete with detailed full-scale dynamic simulations such as OpenSim [22], but is intended to be a reference for such models that aim for rapid adaptability, e.g. in the control of wearable robots.

To approach the question of how such a model can be set up, parameterized and deployed, this paper first describes the experimental setup and the experimental protocol for acquisition of forearm movement data including sEMG recordings of relevant muscles. The resulting dataset based on 31 subjects was published separately [23]. This is followed by a detailed description of the musculoskeletal model of the elbow joint and forearm as a basis for subsequent movement prediction. The section also contains a description of model components as well as of the model parameters. A key point here is the strategy of how to divide the parameters into those that can be determined based on a few anatomical landmarks that can be measured from the outside and those that still need to be adjusted as part of an optimization process. The model was evaluated based on the experiments with 31 subjects described in the data collection section. The methods part of this work concludes with a description of the properties of the chosen error and quality scores in order to compare movement predictions for different experiments and subjects. Finally, the movement prediction results for all subjects and experiments are presented and discussed.

## 2 Methods

In the process of parameter identification for a musculoskeletal elbow joint and forearm model controlled by surface electromyography (sEMG), the first step was the *acquisition of experimental data*. For this, sEMG signals of the muscles involved in forearm actuation and the corresponding elbow angle *θ* were recorded for different subjects and for varying motion sequences (see section 2.1). The experimental data was subsequently used to optimize the parameters of a *musculoskeletal model of the elbow joint* to predict the movement of the forearm (see section 2.2). The majority of the parameters from the model were set to individual values based on prior knowledge. Finally, the last five parameters were optimized by a global and local optimizing process. Since the entire model is based on two muscle heads for each elbow extensor and flexor muscle group, it was assumed that the total torque of all flexors is caused by the *biceps brachii* alone, and the total torque of all extensors is caused by the *triceps brachii* alone (other muscles were not included).

### 2.1 Acquisition of experimental data

#### Subjects

The sEMG data and the corresponding elbow angle *θ* was acquired for *n* = 31 subjects (25 male, 3 female and 3 in none of these categories) while the subjects were performing different motion sequences. 29 subjects chose their right arm as the dominant one, 2 chose the left one as their dominant arm. All subjects were healthy and did not have any prior neural diseases when the experiments were performed. Subjects in this study were randomly labeled with an identification number (ID) starting from 20. Smaller numbers came from pre-experiments to adjust the experimental protocol and were not used. Informed consent was obtained from all subjects in accordance with the policies according to the ethical guidelines of the German Society for Psychology (DGPs) and the German Psychologists Association (BdP), and was approved by the Ethics Committee of the University of Bielefeld (EUB 2017–156 02.08.2017). For each subject, the positions of *acromion* (*ac*) at the shoulder, and the *lateral epicondyle* (*ec*_*l*) at the elbow were defined as reference points to calculate the length *L*_*ac*,*ec*_*l*_ of the upper arm. Furthermore, the positions of the *medial epicondyle* (*ec*_*m*)—again at the elbow—and the *processus styloideus ulnae* (*p*_*su*) at the wrist were defined as reference points to calculate the length *L*_*ec*_*m*, *p*_*su*_ of the forearm down to the wrist. In addition, also the position of the palm was used to calculate a forearm length *L*_*ec*_*m*, *palm*_ that includes the location where the strap loop of the force sensor was held (as will be described below). The latter served as a measure of the length of the lever arm for calculating the elbow torque that occurred due to a measured hand force. An overview of the parameters is given in Table 1.

**Table 1:** Parameters of subjects who participated in the experiments.

	age	height in M	*L*_*ac*,*ec*_*l*_ in m	weight in kg	$F_{hand,max}^{flex}$ in N	$F_{hand,max}^{ext}$ in N	range of *θ* in *deg*
mean	25.3	1.82	0.358	78.7	202.6	167.0	82.7
std	2.76	0.086	0.025	16.5	58.4	58.6	21.2
min	22.0	1.60	0.310	47	115.5	61.3	31.8
max	34.0	1.96	0.400	130	413.5	407.6	130.4

The age is given in whole years. Height and weight were specified by the subjects. The measurement of *F*_*hand*,*max*_ is described in section 2.1. The range of *θ* is calculated according to Eq (33)

#### Determination of maximum isometric muscle force

**Fig 1:**
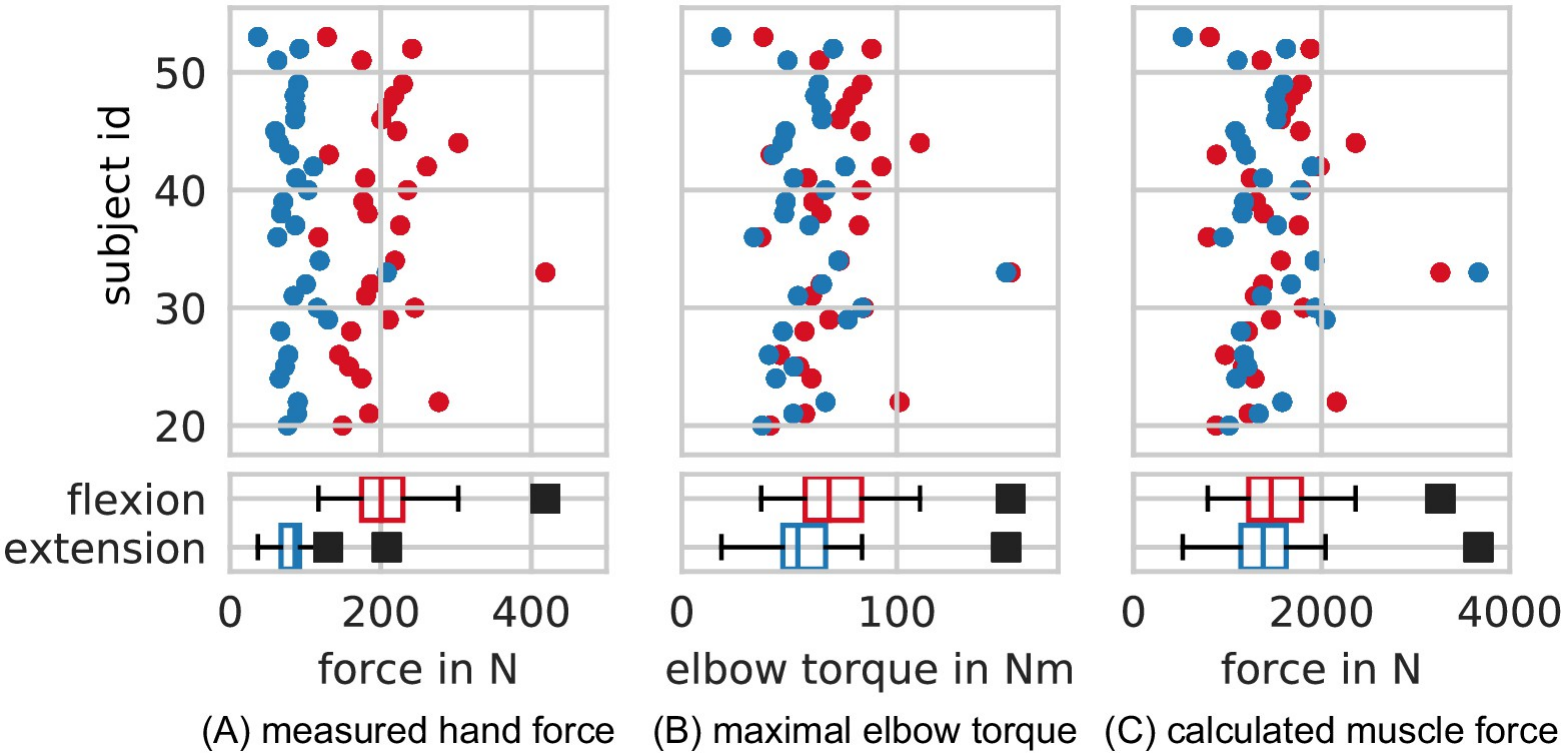
Measured and derived forces and torques of all subjects. (A) shows measured maximal forces *F*_*hand*,*max*_ at subject’s dominant hand for elbow flexion (red) and elbow extension (blue) in the upper panel. Lower panels contains box and whisker plot of data of the upper panel where the box represents the interquartile range (IQR), and the whiskers represent data of 1.5 ⋅ IQR. (B) shows maximal elbow torques that result from the maximal hand forces in (A) when eq:T max is used. (C) contains the maximal muscle forces as calculated based on sec:methods model.

To determine the maximum isometric muscle force, the maximum hand force *F*_*hand*,*max*_ had to be measured first. The maximum hand force was converted into a maximum elbow torque via the corresponding moment arm (forearm length *L*_*ec*_*m*, *palm*_). Later in the process, a maximum muscle force was determined on the basis of a maximum elbow torque and a lever length of the tendon attachment that will be introduced in Eq (11). The maximum hand force was further differentiated into maximum extension force $F_{hand,max}^{ext}$ and maximum flexion force $F_{hand,max}^{flex}$ depending on the direction of the hand force. The former results from the joint action of all muscle (heads) involved in elbow extension (e.g. *triceps* heads) during *maximum voluntary contraction* (*MVC*, see [24, 25]), the latter results from the joint action of all muscle (heads) involved in elbow flexion (e.g. *biceps* heads) during *MVC*. Results of the maximum hand force measurements for all subjects are shown in Fig 1(A).

The system used for the determination of the maximum hand force consisted of an adjustable inelastic strap attached to a piezoelectric force transducer (Typ 9255B, Kistler Instrumente GmbH, Sindelfingen, Germany) which was connected to a charge amplifier (Typ 5011, Kistler Instrumente GmbH, Sindelfingen, Germany). The signal generated by the charge amplifier was recorded with a sample rate of 1kHz using a USB-based A/D-converter card (NI9215, National Instruments corp., Austin, Texas, USA).

To determine the maximum isometric flexion hand force $F_{hand,max}^{flex}$, each subject was instructed to hold the dominant forearm in a horizontal orientation while grabbing the strap via a loop at its end, with the hand oriented such that the thumb pointed upwards. The subjects were then instructed to pull on the strap as strong as possible for a time interval of Δ*t* = 5 s. To ensure that the subject only pulled via the elbow joint, each subject was instructed to minimize shoulder movement while pulling on the strap. The maximum force ($F_{hand,max}^{flex}$) applied during the time interval Δ*t* was saved and the maximum elbow flexion torque ($T_{max}^{flex}$) was computed according to the left side of Eq (1). The data for all subjects are shown in Fig 1(B) where data points for the flexion direction are plotted in red.

$$\begin{array}{c} \underset{\text{(elbow}\;\text{flexion)}}{\underset{︸}{T_{max}^{flex}=F_{hand,max}^{flex}\cdot L_{ec\_m,palm}}},\;\underset{\text{(elbow}\;\text{extension)}}{\underset{︸}{T_{max}^{ext}=F_{hand,max}^{ext}\cdot L_{ec\_m,palm}}}. \end{array}$$ (1)

To determine the maximum isometric hand force $F_{hand,max}^{ext}$ for an elbow extension the subjects were instructed to push as strong as possible with the bottom of the dominant hand placed on the force transducer for a time interval of Δ*t* = 5 s while the maximum force applied was recorded. In this case, subjects were also instructed to hold the dominant forearm in a horizontal orientation and apply force only via the elbow joint and not by e.g. pressing with the upper body. The maximum isometric elbow extension torque $T_{max}^{ext}$ was subsequently computed according to the right side of Eq (1). The data for all subjects are shown in Fig 1(B) where data points for the extension direction are plotted in blue.

#### Surface electromyography (sEMG)

**Fig 2:**
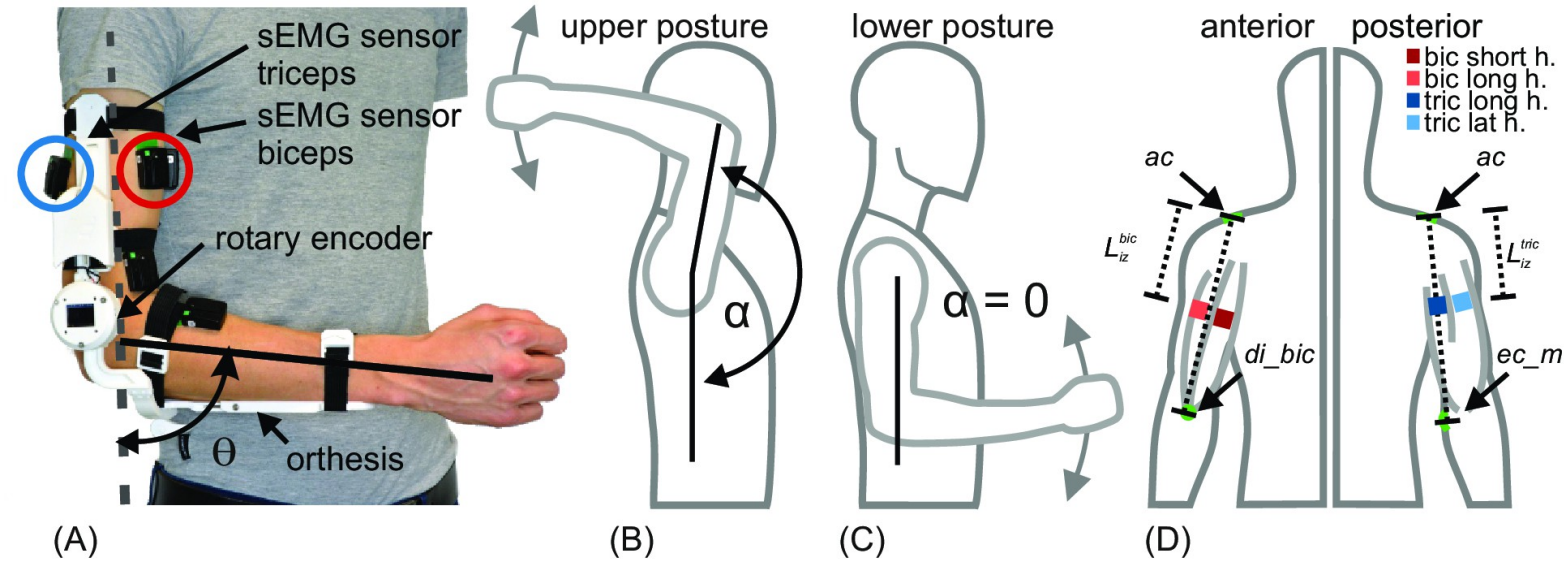
Passive orthosis with sEMG sensors and different experimental postures. (A) shows the passive orthosis and the sEMG sensors placed on the arm. To fix the orthosis to the arm, flexible straps (black) are used. The mounting points and the overall length of the orthosis is customizable. The sEMG sensors were placed onto the *short* and *long head* of the *biceps* and onto the *long* and *lateral head* of the *triceps*. The wrist rotation is in neutral position with the thumb pointing upwards. (A) and (C) show the *lower experimental posture* (*α* approximately 0°). The *upper experimental posture* (*α* almost 180°) is shown in (B). The angle between the upper arm and the body (*α*) is zero when the long axis of the upper arm is pointing towards the ground. In (D) the upper body with the right arm is shown in the coronal plane from an anterior and posterior perspective. Left side shows anterior view with the placement of two *biceps* sEMG sensors in shades of red. Right side shows posterior view with the placement of two triceps sEMG sensor in shades of blue. Lighter tones of red an blue indicate lateral sensor positions to measure the long head of *biceps* and the lateral head of *tricpes*. Darker tones indicate medial sensor position to measure the short head of *biceps* and the long head of *triceps*. This color code is consistent throughout the paper. $L_{iz}^{bic|tric}$ mark the distance from the acromion to the innervation zone.

As input for the muscoskeletal elbow joint and forearm model, sEMG signals of those muscles involved in forearm actuation were used. For the measurement of the respective muscle activity, two wireless sEMG sensors (Delsys Trigno, Delsys, Inc., Boston, MA, USA) were attached to the skin surface above the *biceps brachii* (*bic*: *short head* and *long head*) and two on the skin surface above the *triceps brachii* (*tric*: *long head* and *lateral head* [*medial head* not recorded]). The sEMG sensor is equipped with one sEMG channel with a sampling period of 900 *μS* with 16 bit resolution at a range of 11*mV* and a six axis IMU with a sampling period of 6.75*ms*. Measurements were conducted at a sample rate of 1.111kHz. Before the placement of sensors, the skin was cleaned using isopropanol alcohol. The *acromion* (*ac*), the *medial epicondyle* (*ec*_*m*) and the distal insertion of the *biceps* tendon (*di*_*bic*) were palpated by an experienced experimenter. For the *biceps* heads, the innervation zone lies after 61% of the distance as measured from *ac* to *di*_*bic* [26]. Accordingly, the optimal electrode site is on the muscle belly proximal of the innervation zone ($L_{iz}^{bic}$). For the *triceps* heads, the innervation zone lies after 40% of the distance as measured from *ac* to *ec*_*m* [26]. Again, the optimal electrode site lies proximal of the innervation zone ($L_{iz}^{tric}$). These areas were marked via pen and tape roller accordingly. The individual muscle heads of *biceps* and *triceps* were probed by hand and the innervation zones of the respective muscles, which have to be omitted in sensor placement, were marked via pen and tape roller according to [26]. After the preparation of the skin and the determination of positional placement, sEMG sensors with the included IMU sensors were fixed with double-sided adhesive tape on the skin of the subjects. Fig 2(A) shows the placement of the sensors as well as the passive orthesis used for synchronous measurement of the elbow joint angle *θ*.

The interface between skin and electrodes was evaluated in a preliminary experiment via instructing the subjects to separately contract the flexors and extensors of the upper arm and subsequent visual inspection of the signal quality.

#### Orthetic device for measuring the elbow joint angle

A passive measurement orthosis was used to measure the elbow angle *θ* synchronously with sEMG recordings while the subjects were performing different motion sequences (Fig 2). The measurement orthosis was custom designed and 3D-printed in-house from PLA plastic. The attachment locations on the body and the overall length of the orthosis can be adjusted to different arm sizes. The elbow angle is determined using a 10-bit magnetic rotary position encoder (AS5043, ams AG, Premstaetten, Austria) which is integrated in the joint of the orthosis. Thus, it is aligned to the rotary axis of the elbow joint of the subject. The analog output of the rotary encoder is fed into a Trigno Analog Adapter (Delsys, Inc., Boston, MA, USA) to allow for synchronous recording of the elbow joint angle and the sEMG signals. Since the limbs of the measurement orthosis were not necessarily aligned and parallel to the long axes of the forearm and upper arm, a calibration was performed by the supervisor during the initial phase of the experiment.

#### Experimental paradigm

The subjects were instructed to perform different motion sequences while sEMG signals of the muscles involved in forearm actuation and the elbow joint angle were recorded. The motion sequences consisted of periodic movements of the dominant forearm for two different postures of the respective upper arm (see Fig 2(B) and 2(C)) with three different weights held by the subject. For each posture, subjects were instructed to align the longitudinal axis of the forearm orthogonal to the longitudinal axis of the upper arm resulting in an initial elbow angle of *θ*_0_ = 90° (see also Fig 2(A)). In the *lower posture* subjects were instructed to hold the upper arm vertically pointing downwards and in the *upper posture* the upper arm was held vertically pointing upwards. In contrast to the *lower posture*, the *upper posture* varies among the subjects due to different flexibility in the subjects’ shoulders. To address this inconsistency, the IMU of the Trigno sensor was used to determine the orientation of the upper arm. For each posture a weight held by the subject was varied (*w* = [2kg, 4kg]) resulting in four different posture-weight combinations that correspondingly led to different loads on the involved muscle groups. After the initial static orientation of the arm for Δ*t* = 5 s, the subjects were instructed to move the forearm back and forth rhythmically about the axis of the elbow joint at as constant angular velocity as possible, resulting in a sine-like modulation of the elbow joint angle. The target range of the movement was ±45° around the initial position. To define the frequency of the sinusoidal elbow-joint rotation, a metronome was used to provide a visual and auditory hint to the subjects. For each posture-weight combination experiments were performed at [0.25Hz (slow) and 0.5Hz (fast)]. After Δ*t* = 30 s of dynamic movement subjects were instructed that the movement should come to an end naturally, implying that the subject could finish the current move and stopped close to the initial position *θ*_0_. After each trial the subjects were allowed to rest for at least one minute. After the resting phase, the trials were repeated with a different posture-weight combination. The order was as follows: fast 2 kg, fast 4 kg, slow 2 kg, slow 4 kg—first in the *lower posture*, than in the *upper posture*.

**Fig 3:**
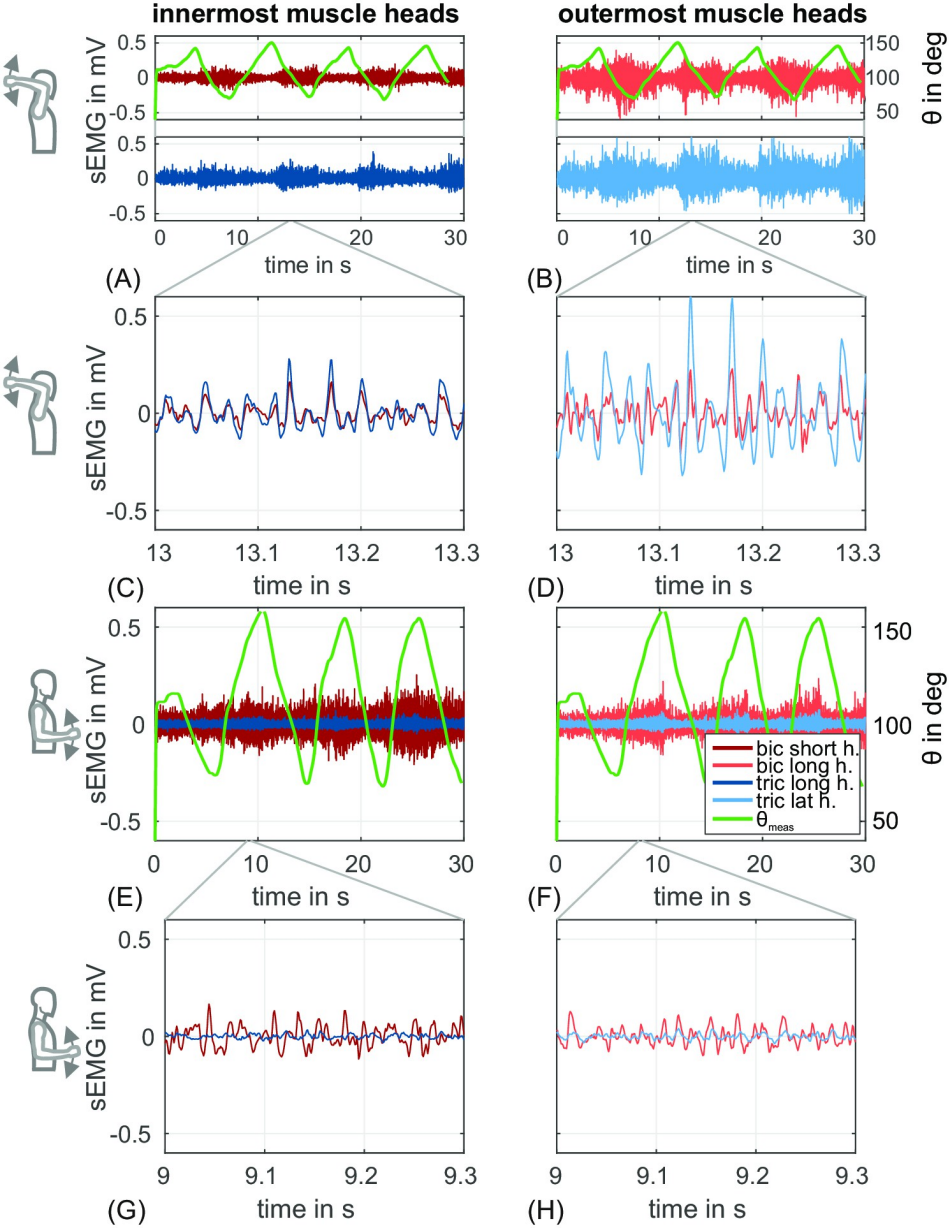
Raw values of sEMG signals and elbow joint angle of a single subject. (A,B,C,D) show sEMG signals for the *upper posture*. (E,F,G,H) show sEMG signals for the *lower posture*. *Biceps* sEMGs are shown in shades of red and *triceps* sEMGs in shades of blue. Left column (A,C,E,G) show sEMG signals from innermost muscle heads in darker shades and right column (B,D,F,H) show sEMG signals from outermost muscle heads in lighter shades of the respective color. On the right axis the elbow angle (*θ*) is plotted in green. *θ* = 0° means that the arm is fully extended. (C,D,G,H) each show a time frame of 0.3 s from the respective signals arranged above.

Fig 3 gives an exemplary overview of the time behavior for the amplitudes of the data recorded in the experiments described above to serve as input into the predictive musculoskeletal model described in section 2.2. The data in the figure were collected from one single subject. sEMG signals from the two *biceps* heads are shown in shades of red (light red for the *long head* [lateral location in upper arm] and dark red for the *short head* [innermost location on the upper arm]). sEMG signals from two *triceps* heads are shown in shades of blue (light blue for the *lateral head* [lateral location on the upper arm] and dark blue for the *long head* [innermost location on the upper arm]). Upper posture (Fig 3(A)–3(D)) and lower posture (Fig 3(E)–3(H)) are indicated via symbolic figures on the left.

sEMG signals differ in the variation of the time behavior for the amplitudes between subjects and also characteristics of muscle utilization (co-contraction, reciprocal contraction) are different for the same motion between subjects.

### 2.2 Musculoskeletal model of the elbow joint

**Fig 4:**
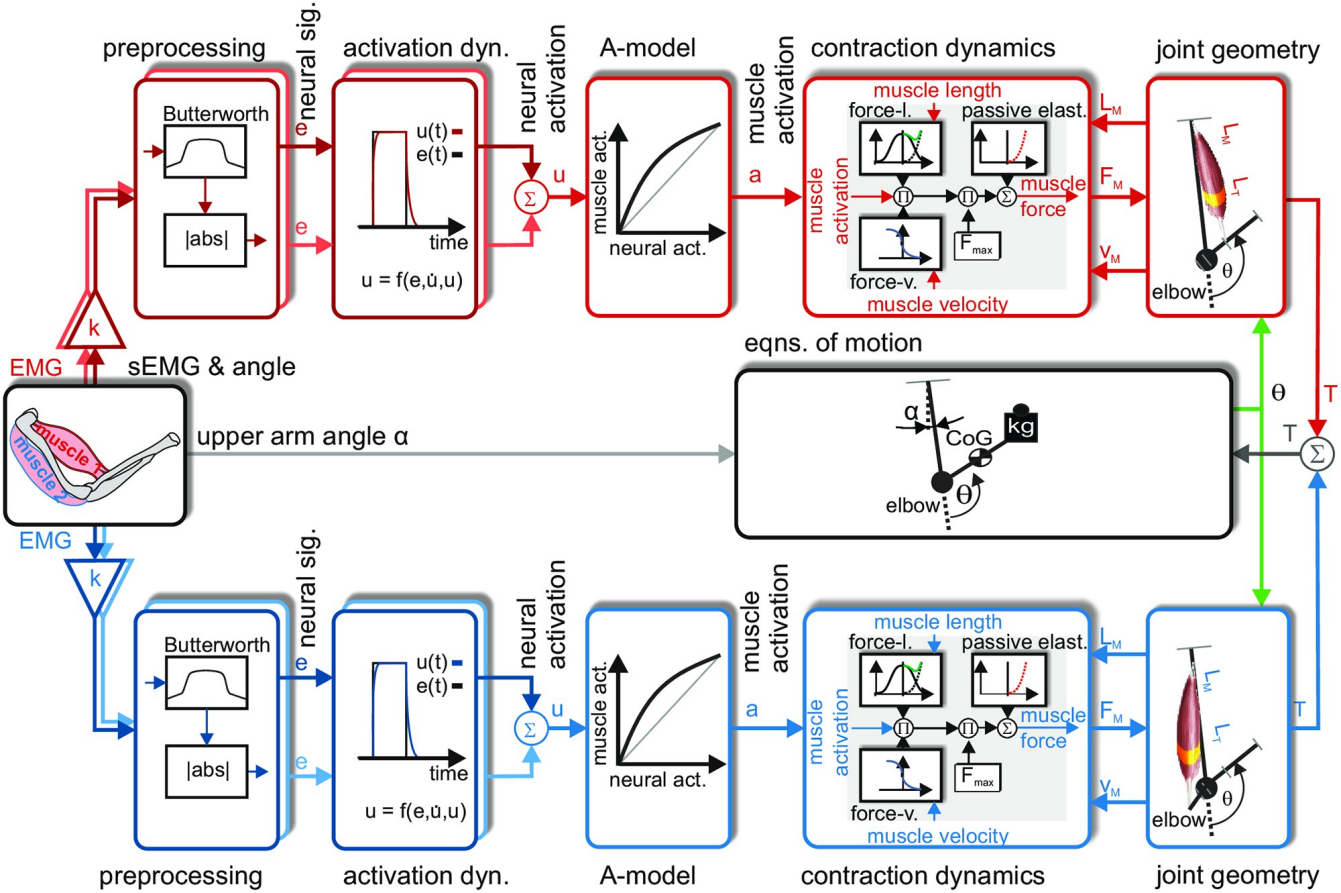
Block diagram of musculoskeletal model of elbow joint. The general signal flow in the diagram is from left to right. Signal sources of the model are shown on the left side and contain sEMG signals and the upper arm angle *α* (relevant for the arm posture, see Fig 2(B) and 2(C)). sEMG signals of muscle heads involved in elbow flexion, in this work the two heads of the *biceps brachii*, are depicted in the upper row in shades of red. sEMG signals of muscles heads involved in elbow extension, in this work two of the three heads of the *triceps brachii*, are depicted in the lower row in shades of blue. Darker shades of a color represent the innermost head of the respective muscle, lighter shades of a color represent the outermost head of the respective muscle. After *preprocessing* of the sEMG signals, the resulting *neural signals* were fed into the *activation dynamics*, added up and served as input to the A-model. The output of the A-model, which is the *muscle activation*, was fed into the submodel for the *contraction dynamics*. Its output, which is the respective muscle force, was applied to the *joint geometry* submodel which calculates the respective torque. Resulting torques were summed up and fed into the *equations of motion* submodel where the resulting movement of the forearm was calculated. The main output of the overall model is the joint angle *θ* of the elbow (see Fig 2(A)) which was further used to calculate current muscle velocities and lengths.

The model, shown in Fig 4, was used to simulate the behavior of components of the musculoskeletal system of elbow and forearm using sEMG signals of the respective muscles to predict the forearm movement. The overall structure of the simulation model is derived from the biochemical and biomechanical target system. Hence, the model used comprises both the electrophysiological properties of muscle activation and the mechanical properties underlying forearm actuation.

The four sEMG signals recorded from each of the two different heads of the *biceps* and *triceps* served as an input to the model and were *preprocessed* separately resulting in a muscle-specific *neural activation*. In the model, corresponding neural activations of each muscle head are summed up and the sum is used as input for a non-linear submodel (so called *A-model*, [8]). For each antagonistic muscle activation provided, here the *biceps* and *triceps*, two independent *contraction dynamics* and *joint geometries* are used to simulate muscular force and muscular torque generation. In the context of this work, the *biceps* acted as the only flexor and the *triceps* as the only extensor in the simulation of *musculoskeletal mechanics*. The *equation of motion* submodel contains the musculoskeletal mechanics and was used to calculated the elbow joint angle and is driven by the torques generated by the involved muscles. The corresponding muscle velocities and muscle lengths obtained from the muscular mechanics are fed back to drive the contraction dynamics submodel. The described signal flow is shown in Fig 4. Details of the submodels will be introduced below.

#### sEMG prepossessing

The sEMG signals measured at the two heads of the *biceps brachii* and the *triceps brachii*, respectively, served as inputs for the musculoskeletal model of the elbow joint. Each of the sEMG signals was preprocessed, i.e. amplified, filtered, and rectified, to eliminate interfering signals and to raise the input signal to a level that was comparable for different subjects.

At first, the input was amplified by a factor *k*. This factor was chosen in the optimization process such that the range between no contraction and a maximum voluntary contraction leads to a signal interval between minus one and one, or rather between zero and one for the following activation dynamics (after filtering and rectification). The value of *k* is also related to the resistance of the electrode-skin-interface and may drift over time. Signal filtering was achieved with a 4th order Butterworth bandpass with cutoff frequencies of *f*_*low*_ = 4Hz and *f*_*high*_ = 400 Hz [27] to reduce noise. Rectifying the signal, as introduced in [7], resulted in the neural activation *e* which ranges mostly between zero and one. This range can be interpreted as the activation level of the neurons. All parameters of this paragraph are summarized in S1 Table.

#### Activation dynamics

The activation of a muscle as a result of neural excitation (recorded as sEMG signals) was modelled in the *activation dynamics* submodel. This submodel represents the electrochemical conversion of neural signals (spike trains on the muscle fibre) into the release of Ca^2+^ ions in the muscle sarcomeres, ultimately enabling mechanical contraction of the sarcomeres and—as a consequence—of the muscle as described e.g. by Zajac or Buchanan and co-workers [7, 8]. In terms of signal flow, the *activation dynamics* submodel converts the neural signal *e* into the *neural activation* *u*. It was formulated as a non-linear, first order differential equation [7] as shown in Eq (2).

$$\begin{array}{c} \frac{du(t)}{dt}+[\frac{1}{\tau_{act}}\cdot \left(\beta +\left[1-\beta \right]e\left(t\right)\right)]\cdot u\left(t\right)=(\frac{1}{\tau_{act}})\cdot e\left(t\right)\\ 0<\beta =const.<1 \end{array}$$ (2)

Here, *τ*_*act*_ is the time constant and *β* is a dimensionless parameter which essentially sets the time response when the activation is suspended as compared to the beginning of the activation. *τ*_*act*_ (17.3 ms) and *β* (0.35) were set, such that the step response matches a reference step response as formulated in [28]. Neural activation of one muscle were summed up and fed into the A-model [8]. The A-model represents a nonlinear behavior when the *neural activation u*(*t*) is translated into the *muscle activation a*(*t*) (which results from a nonlinear relationship between stimulation frequency and force [8]). The respective formulation for the A-model is shown in Eq (3).

$$\begin{array}{c} a\left(t\right)=\frac{e^{A\cdot u(t)}-1}{e^{A}-1} \end{array}$$ (3)

Eq (3) contains only one parameter *A* to shape the nonlinearity which lies in the interval −0.001 ≤ *A* ≤ −3 [8]. A coarse search was initially performed on different subjects and resulted in a value of *A* = −0.25. All parameters of this paragraph are summarized in S2 Table.

#### Contraction dynamics

The *contraction dynamics* submodel models the relationship between muscle activation *a*(*t*) and muscle force *F*_*M*_(*t*). Besides activation, the muscle force also depends on the current muscle length *L*_*M*_(*t*), and the current muscle contraction or muscle relaxation speed *v*_*M*_(*t*) [7]. The length dependence of the muscle force is divided into an activation-dependent (active) component *F*_*L*_(Δ*L*_*M*_(*t*)) and an activation-independent (passive) component *F*_*p*_(Δ*L*_*M*_(*t*)), where the length dependence is represented as a deviation Δ*L*_*M*_(*t*) from the resting (optimal) length *L*_*M*,0_ of the muscle:

$$\begin{array}{c} \Delta L_{M}\left(t\right)=L_{M}\left(t\right)-L_{M,0}. \end{array}$$ (4)

Based on activation, muscle length deviation from resting length, and muscle contraction (relaxation) velocity, the muscle force can be formulated as

$$\begin{array}{c} F_{M}\left(a\left(t\right),\Delta L_{M}\left(t\right),v_{M}\left(t\right)\right)=F_{\text{max}}\cdot (\underset{\text{active}\;\text{muscle}\;\text{force}}{\underset{︸}{a\left(t\right)\cdot F_{L}\left(\Delta L_{M}\left(t\right)\right)\cdot F_{v}\left(v_{M}\left(t\right)\right)}}+\underset{\text{passive}\;\text{muscle}\;\text{force}}{\underset{︸}{F_{p}\left(\Delta L_{M}\left(t\right)\right)}}). \end{array}$$ (5)

With *F*_max_ being the maximum isometric force of the respective muscle (*biceps* or *triceps*) as later described in Eq (28).

The length-dependent part *F*_*L*_(Δ*L*_*M*_(*t*)) (force-length-function) of the active muscle force reflects the amount of overlap of *actin* and *myosin* filaments in the muscle *sarcomeres* to potentially form cross-bridges. Deviations from the (optimal) resting length of the muscle lead to smaller forces. According e.g. to Geyer and co-workers [29], the force-length-function can be formulated as

$$\begin{array}{c} F_{L}\left(\Delta L_{M}\left(t\right)\right)=\text{exp}\left(c\cdot {\frac{\Delta L_{M}\left(t\right)}{L_{M,0}\cdot w}}^{3}\right)\;\text{,}\;0<F_{L}\left(\Delta L_{M}\left(t\right)\right)\leq 1\;\forall \;\Delta L_{M}\left(t\right) \end{array}$$ (6)

where *w* is a parameter to control the width of the bell-shaped force-length-function. According to [7, 8, 16, 30] this parameter was set to a standard value of *w* = 0.5 to reflect a maximum length increase/decrease of the muscle length by 50% w.r.t. the resting length. The shape factor *c* was set to *c* = ln0.05 to fullfill *F*_*L*_(Δ*L*_*M*_(*t*) = ±*w* ⋅ *L*_*M*,0_) = 0.05 (i.e. a force decline down to 5% of the maximum force at a length deviation of 50% from the resting length [29]), as well es the value range condition given in Eq (6).

The velocity-dependent part *F*_*v*_(*v*_*M*_(*t*)) (force-velocity-function) of the active muscle force was formulated in a two-part equation as proposed e.g. by Geyer et al. [29] and shown in Eq (7).

$$\begin{array}{c} F_{v}\left(v_{M}\left(t\right)\right)=\{\begin{array}{cc} \frac{v_{M,\text{max}}-v_{M}\left(t\right)}{v_{M,\text{max}}+K_{v}\cdot v_{M}\left(t\right)} & \text{if}\;v_{M}\left(t\right)<0\;\text{(shortening,}\;\text{concentric)}\\ N+\left(N-1\right)\frac{v_{M,\text{max}}-v_{M}\left(t\right)}{7.56\cdot K_{v}\cdot v_{M}\left(t\right)-v_{M,\text{max}}} & \text{if}\;v_{M}\left(t\right)\geq 0\;\text{(lengthening,}\;\text{excentric)} \end{array} \end{array}$$ (7)

The shortening part of Eq (7) describes the effect formulated by Hill [13] for active muscle shortening. The lengthening part follows a formulation by Aubert [31] with *N* as a dimensionless maximum (force) value reached at *v*_*M*_(*t*) = *v*_*M*,max_. Thelen has shown that *N* varies with age [17] from *N* = 1.4 for young adults to *N* = 1.8 for old adults. Since the mean age of the subjects in this study was 25.3 years (Table 1), N was set to *N* = 1.4. The shape factor *K*_*v*_ changes the curvature of the lengthening part and was set to *K*_*v*_ = 5 [29–31]. The maximum velocity was set to *v*_*M*,max_ = 10 ⋅ *L*_*M*,0_ ⋅ s^−1^ [17, 32].

The passive part of the muscle force is generated by an non-linear elasticity (attributed to the elastic protein *titin*) which acts in parallel to the contractile elements (*actin myosin* complex) in the *sarcomere* and which accounts for the return of the unactivated muscle to its resting state [7]. Zajac modelled this as shown in Eq (8) [7].

$$\begin{array}{c} F_{p}\left(\Delta L_{M}\left(t\right)\right)=\{\begin{array}{cc} K_{p}\cdot {\left(L_{M}\left(t\right)-L_{M,0}\right)}^{2} & \;\text{if}\;L_{M}\left(t\right)\geq L_{M,0}\\ 0 & \;\text{otherwise} \end{array} \end{array}$$ (8)

The factor *K*_*p*_ sets the slope of the quadratic function. Values for *K*_*p*_ vary between *K*_*p*_ = 3 [32–34] to *K*_*p*_ = 4 [35]. Thelen has shown that values for *K*_*p*_ vary with age between *K*_*p*_ = 2.78 for young adults to *K*_*p*_ = 4 for older adults [17] (Because Thelen uses a different formulation for passive elasticity, the values were converted accordingly.). Since the mean age of the subjects in this study was 25.3 years (Table 1), *K*_*p*_ was set to the lower boundary *K*_*p*_ = 2.78. All parameters of this paragraph are summarized in S3 Table.

#### Musculoskeletal mechanics

As the *biceps* and *triceps* muscles contract, their forces are propagated to the bones of the upper and forearm via the insertion points of the respective tendons, causing torques in the elbow joint. The tendon is generally assumed to be a non-linear spring with a high stiffness in the linear region [36]. At the maximal force, the elongation is about 3% [7]. As the experiment conducted here is far from maximum force, the lengthening of the tendon was neglected. With this assumption, Eq (9) is valid.

$$\begin{array}{c} L_{M}^{\text{bic}|\text{tric}}=L_{MTC}^{\text{bic}|\text{tric}}-L_{T}^{\text{bic}|\text{tric}} \end{array}$$ (9)

**Fig 5:**
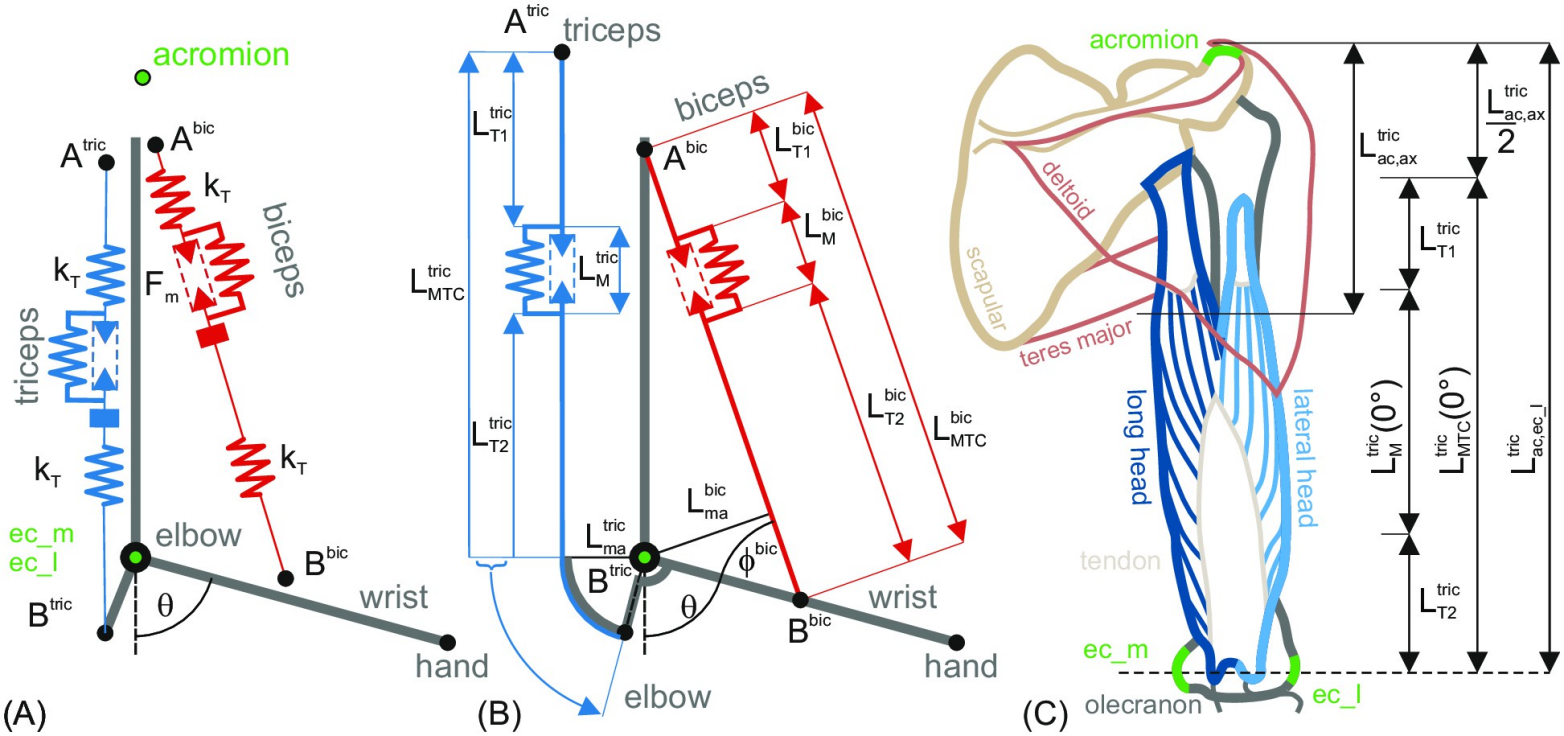
Mechanical model of the arm with dimensions and reference points. (A) Schematic depiction of upper arm and forearm with elbow joint together with *biceps* and *triceps brachii* muscle models. Mechanical support structures representing the bones are plotted as bold lines. Three externally palpable landmarks (acromion, *ac* as well as *medial* and *lateral epicondyles*, *ec*_*m* and *ec*_*l*) are illustrated in green. The hand/wrist marks the lateral end of the forearm. The musculotendon complexes (*MTC*) for both muscles were depicted schematically as spring-mass systems with separate tendon elasticities. The *MTCs* were spanned between the respective insertion points (*A*^bic|tric^, *B*^bic|tric^). The angle of the elbow joint *θ* was defined as 0 for the fully extended arm and positive for counter-clockwise flexion movements. (B) shows a reduced model version of (A) in which most insertion points were placed on the support structures and the tendons were neglected. The graphic also shows the definition of the length specifications. The *MTC* of the *triceps* was guided over a pulley wheel with constant radius. (C) *Posterior* view of upper arm with two heads of *biceps brachii* in shades of blue and the three externally palpable landmarks in green. Landmarks were used to estimate the depicted length measures (for details see text).

With an inelastic tendon, the velocity of the tendon (*v*_*T*_) and the velocity of the muscle (*v*_*M*_) are equal. The simplified model of the mechanical configuration of the elbow joint in Fig 5(B) was the basis for the following considerations. For the sake of simplicity, single insertion points *A*^bic^ and *A*^tric^ were assumed for the upper tendons of the *biceps* and *triceps*. Each of the two designators also stands for the distance of the respective insertion point to the *medial epicondyle* (*ec*_*m*). Also for the lower tendons of *biceps* and *triceps*, single insertion points *B*^bic^ and *B*^tric^ were assumed at the forearm. Again, each of the designators represents the distance of the respective insertion point to the *medial epicondyle*. As shown Fig 5(B), the insertion point of the lower *triceps* tendon is located at the osseous structure extension of *ulna* called *olecranon*.

In addition to the elbow angle *θ*(*t*), for the *biceps* the auxiliary angle *ϕ*^bic^(*θ*(*t*)) was introduced which is the angle between the *biceps* muscle and the forearm. With changing elbow angle *θ*(*t*), also the *biceps* moment arm $L_{\text{ma}}^{\text{bic}}(\phi^{\text{bic}}(\theta (t)))$ and the *triceps* moment arm $L_{\text{ma}}^{\text{tric}}\left(\theta \left(t\right)\right)$ change. As the muscle forces are always positive, the signs of the moment arms were chosen such that the positive sign of the *biceps* moment arm $L_{\text{ma}}^{\text{bic}}(\phi^{\text{bic}}(\theta (t)))$ leads to a positive torque and thus to a counterclockwise rotation of the elbow joint and the negative sign of the *triceps* moment arm $L_{\text{ma}}^{\text{tric}}\left(\theta \left(t\right)\right)$ to a negative torque and thus to a clockwise rotation. With the distance from upper insertion point of the upper *biceps* tendon *A*^bic^ to medial epicondyle (which represents the elbow axis) and the distance from medial epicondyle to the insertion point of the lower *biceps* tendon *B*^bic^, the current length of the *biceps* muscular tendon complex $L_{MTC}^{\text{bic}}(\theta \left(t\right),\phi^{\text{bic}}(\theta (t)))$ and the respective moment arm $L_{\text{ma}}^{\text{bic}}(\phi^{\text{bic}}(\theta (t)))$ can be calculated. According to [37], the angle *ϕ*^bic^(*θ*(*t*)) for the calculation of the muscle moment arms can be calculated as follows:

$$\begin{array}{c} \phi^{\text{bic}}\left(\theta \left(t\right)\right)\{\begin{array}{cc} \text{atan2}(\frac{A^{\text{bic}}\cdot \text{sin}\left(\theta \left(t\right)\right)}{B^{\text{bic}}+A^{\text{bic}}\cdot \text{cos}\left(\theta \left(t\right)\right)}) & \text{if}\;\theta \left(t\right)>\theta_{min}^{\text{bic}}\\ \text{atan2}(\frac{A^{\text{bic}}\cdot \text{sin}\left(\theta_{min}\right)}{B^{\text{bic}}+A^{\text{bic}}\cdot \text{cos}\left(\theta_{min}\right)}) & \text{otherwise}\; \end{array} \end{array}$$ (10)

The moment arm of the *biceps* was calculated according to:

$$\begin{array}{c} L_{\text{ma}}^{\text{bic}}(\phi^{\text{bic}}(\theta (t)))=B^{\text{bic}}\cdot \text{sin}\left(\phi^{\text{bic}}\left(\theta \left(t\right)\right)\right) \end{array}$$ (11)

And finally, the length of the muscle tendon complex resulted in:

$$\begin{array}{c} L_{MTC}^{\text{bic}}(\theta \left(t\right),\phi^{\text{bic}}(\theta (t)))=\frac{B^{\text{bic}}+A^{\text{bic}}\cdot \text{cos}\left(\theta \left(t\right)\right)}{\text{cos}(\phi^{\text{bic}}(\theta (t)))} \end{array}$$ (12)

As can be seen in Fig 5(B), the length *B*^bic^ is equal to the maximum length of the moment arm $L_{\text{ma}}^{\text{bic}}(\phi^{\text{bic}}(\theta (t)))$. Murray et al. conducted a study on the courses of elbow muscles’ moment arms [38] from which *B*^bic^ = 0.0472m was adopted. As the length *A*^bic^ is not directly accessible, it was derived from the length of the upper arm *L*_*ac*,*ec*_*l*_ by using a fixed ratio ${\bar{r}}_{L_{ac,ec\_l},A}^{\text{bic}}=0.9$ derived from [39] (*A*^bic^ = 0.293m and *L*_forearm_ = 0.416m) and [40] (*L*_*ac*,*ec*_*l*_ = 0.618 ⋅ *L*_forearm_). *A*^bic^ can then be calculated for each subject individually according to Eq (13).

$$\begin{array}{c} A^{\text{bic}}=\frac{L_{ac,ec\_l}}{{\bar{r}}_{Lac,ec\_l,A}^{\text{bic}}} \end{array}$$ (13)

The individual length *A*^bic^ was further used to derive the individual resting length $L_{MTC,0}^{\text{bic}}$ of the *biceps* muscle tendon complex for each subject in Eq (14). For this, an average ratio ${\bar{r}}_{LMTC0,A}^{\text{bic}}$ has to be found. This requires a mean value for the upper *biceps* insertion point which is given by Winters and Stark with ${\bar{A}}^{\text{bic}}=0.335\;\text{m}$ [41]. Furthermore, the mean length of the muscle tendon complex of the *biceps* at optimal muscle length is also given by Winter and Stark with ${\bar{L}}_{MTC,0}^{\text{bic}}=0.36\;\text{m}$. Using these two values results in mean ratio ${\bar{r}}_{LMTC0,A}^{\text{bic}}=1.07$. With this, $L_{MTC,0}^{\text{bic}}$ can be calculated individually as follows:

$$\begin{array}{c} L_{MTC,0}^{\text{bic}}=A^{\text{bic}}\cdot {\bar{r}}_{LMTC0,A}^{\text{bic}}\;\text{with}\;\;{\bar{r}}_{LMTC0,A}^{\text{bic}}=\frac{{\bar{L}}_{MTC,0}^{\text{bic}}}{{\bar{A}}^{\text{bic}}}=1.07 \end{array}$$ (14)

The resting length $L_{MTC,0}^{\text{bic}}$ of the muscle tendon complex from Eq (14) contains the resting length of the muscle $L_{M,0}^{\text{bic}}$ as well as the length of the tendon $L_{T}^{\text{bic}}$. This condition is also applied to the *triceps*. To calculate the resting length of the muscle $L_{M,0}^{\text{bic}|\text{tric}}$ from the resting length of the muscle tendon complex $L_{MTC,0}^{\text{bic}|\text{tric}}$ for each subject, again a fixed mean ratio ${\bar{r}}_{LMTC0,LM0}^{\text{bic}|\text{tric}}$ was used. It was calculated based on average values as given in Winters and Stark [41]. These values are the average length of the muscle tendon complex ${\bar{L}}_{MTC,0}^{\text{bic}}=0.36\;\text{m}$, and ${\bar{L}}_{MTC,0}^{\text{tric}}=0.28\;\text{m}$, as well as the average length of the resting *biceps* and *triceps* muscle ${\bar{L}}_{M,0}^{\text{bic}}=0.145\;\text{m}$, and ${\bar{L}}_{M,0}^{\text{tric}}=0.08\;\text{m}$. This results in a ${\bar{r}}_{LMTC0,LM0}^{\text{bic}}=2.48$ and ${\bar{r}}_{LMTC0,LM0}^{\text{tric}}=3.5$.

$$\begin{array}{c} L_{M,0}^{\text{bic}|\text{tric}}=\frac{L_{MTC,0}^{\text{bic}|\text{tric}}}{{\bar{r}}_{LMTC0,LM0}^{\text{bic}|\text{tric}}}\;\text{with} \end{array}$$ (15)

$$\begin{array}{c} {\bar{r}}_{LMTC0,LM0}^{\text{bic}}=2.48\;{\bar{r}}_{LMTC0,LM0}^{\text{tric}}=3.5 \end{array}$$ (16)

The resulting individual resting muscle length was used in the contraction dynamics (Eqs (6) and (8)).

The origin of the *triceps* long head is at the *scapular* and the origin of the respective lateral head is at the *humerus*. The goal was to calculate a combined *A*^tric^ for the two heads for each individual subject based on the measured individual length *L*_*ac*,*ec*_*l*_ and a mean ratio ${\bar{r}}_{Lac,ec\_l,A}^{\text{tric}}$ as shown in Eq (18). For the latter, an average ${\bar{A}}^{\text{tric}}$ had to be determined. For that, it was assumed that the origin of the *triceps A*^tric^ can be found halfway between the *acromion* and the axillary fold. A mean distance between the *acromion* and the axillary fold is given by Gordon and coworkers [42, p. 346] (based on the ANSUR I dataset, [43]) with ${\bar{L}}_{ac,ax}=0.121\;\text{m}$ for male subjects and with ${\bar{L}}_{ac,ax}=0.099\;\text{m}$ for female subjects. The corresponding mean distance between *acromion* and *lateral epicondyle* is given with ${\bar{L}}_{ac,ec\_l}=0.340\;\text{m}$ for male subjects and ${\bar{L}}_{ac,ec\_l}=0.311\;\text{m}$ for female subjects [42, p. 80]. The resulting ${\bar{A}}^{\text{tric}}$ was calculated as follows:

$$\begin{array}{c} {\bar{A}}^{\text{tric}}={\bar{L}}_{ac,ec\_l}-\frac{{\bar{L}}_{ac,ax}}{2} \end{array}$$ (17)

This resulted in a mean distance between elbow rotation axis and *triceps* origin for male subjects of ${\bar{A}}^{\text{tric}}=0.28\;\text{m}$ and for female subjects of ${\bar{A}}^{\text{tric}}=0.26\;\text{m}$. This mean distance together with the mean value of ${\bar{L}}_{ac,ec\_l}$ was further used to calculate the mean ratio ${\bar{r}}_{Lac,ec\_l,A}^{\text{tric}}$. For male subjects this resulted in ${\bar{r}}_{Lac,ec\_l,A}^{\text{tric}}=1.22$ and for female in ${\bar{r}}_{Lac,ec\_l,A}^{\text{tric}}=1.20$.

$$\begin{array}{c} A^{\text{tric}}=\frac{L_{ac,ec\_l}}{{\bar{r}}_{Lac,ec\_l,A}^{\text{tric}}}\;\text{with}\;\;{\bar{r}}_{Lac,ec\_l,A}^{\text{tric}}=\frac{{\bar{L}}_{ac,ec\_l}}{{\bar{A}}^{\text{tric}}} \end{array}$$ (18)

The lower tendon of the *tricpes* ends at the *olecranon*. Here, it was assumed that the *olecranon* and the the *medial epicondyle* are at the same height in the *sagittal* plane if the elbow is fully extended [44]. Therefore, it can be assumed that $L_{MTC}^{\text{tric}}\left(0^{\circ }\right)=A^{tric}$ applies which leads to:

$$\begin{array}{c} L_{M}^{\text{tric}}\left(0^{\circ }\right)=\left(A^{\text{tric}}-L_{T}^{\text{tric}}\right) \end{array}$$ (19)

In the movement range of the elbow [0°…120°], the length of the *triceps* varies from $0.54\cdot L_{M,0}^{\text{tric}}$ to $1.41\cdot L_{M,0}^{\text{tric}}$ [16]. For the case of a fully extended elbow, Eq (20) can be used to calculate the resting length of the *triceps* muscle:

$$\begin{array}{c} L_{M,0}^{\text{tric}}=\frac{L_{M}^{\text{tric}}\left(0^{\circ }\right)}{0.54} \end{array}$$ (20)

With Eq (16) rearranged to

$$\underset{L_{MTC,0}^{\text{tric}}}{\underset{︸}{L_{M,0}^{\text{tric}}+L_{T}^{\text{tric}}}}={\bar{r}}_{LMTC0,LM0}^{\text{tric}}\cdot L_{M,0}^{\text{tric}}$$ (21)

$$L_{T}^{\text{tric}}={\bar{r}}_{LMTC0,LM0}^{\text{tric}}\cdot L_{M,0}^{\text{tric}}-L_{M,0}^{\text{tric}}\;,$$ (22)

inserted into Eq (19), and using Eq (20) LM 54 allows to calculate the resting length of the *triceps* muscle according to:

$$L_{M,0}^{\text{tric}}=(A^{\text{tric}}-r_{LMTC0,LM0}^{\text{tric}}\cdot L_{M,0}^{\text{tric}}+L_{M,0}^{\text{tric}})/0.54$$ (23)

$$L_{M,0}^{\text{tric}}=\frac{A^{\text{tric}}}{\left(r_{LMTC0,LM0}^{\text{tric}}-0.46\right)}$$ (24)

As the moment arm $L_{\text{ma}}^{\text{tric}}$ is nearly constant over the elbow angle [14], it was modeled as a pulley with constant radius. Before running the simulation, the moment arm $L_{\text{ma}}^{\text{tric}}$ can be calculated based on the maximum shortening/ lengthening range of the muscle according to:

$$\begin{array}{c} L_{\text{ma}}^{\text{tric}}=\left(1.41-0.54\right)\cdot L_{M,0}^{\text{tric}}\cdot \frac{1}{2\pi }\cdot 3 \end{array}$$ (25)

Note that the maximum shortening/ lengthening range in [16] is specified for a third of a revolution.

The calculated values for the the resting length ($L_{M,0}^{\text{tric}}$, Eq (24)) and the moment arm ($L_{\text{ma}}^{\text{tric}}$, Eq (25)) were used to calculate $L_{M}^{\text{tric}}$ during simulation:

$$\begin{array}{c} L_{M}^{\text{tric}}\left(\theta \right)=\underset{\text{minimal}\;\text{muscle}\;\text{length}}{\underset{︸}{0.54\cdot L_{M,0}^{\text{tric}}}}+L_{\text{ma}}^{\text{tric}}\cdot \theta +L_{\text{offset}}^{\text{tric}} \end{array}$$ (26)

The only unknown parameter $L_{\text{offset}}^{\text{tric}}$ compensates inaccuracies and is subject to the optimization process.

The length of the muscle and the velocity were fed back to the contraction dynamics (cf. Fig 4). The force from the contraction dynamics ($F_{M}^{\text{bic}|\text{tric}}$) was multiplied by the moment arm ($L_{\text{ma}}^{\text{bic}|\text{tric}}$) to get the elbow torque as generated by the muscle.

$$\begin{array}{c} T=\underset{\text{biceps}\;\text{torque}}{\underset{︸}{F_{M}^{\text{bic}}\cdot L_{\text{ma}}^{\text{bic}}}}+\underset{\text{triceps}\;\text{torque}}{\underset{︸}{F_{M}^{\text{tric}}\cdot \left(-L_{\text{ma}}^{\text{tric}}\right)}} \end{array}$$ (27)

The direction of the angle and the geometric dimensions of the arm are shown in Fig 5(B).

#### Equation of motion

For the simulation of the mechanical system, the masses and centers of gravity (cog) of the arm segments were required. The masses of forearm and hand were based on findings by Bernstein [45]. For the forearm mass *m*_forearm_ a portion of 1.82% of the body mass for both women and men is given. The hand mass *m*_hand_ accounts for 0.7% of the body mass for men and for 0.55% of the body mass for women. The center of gravity (cog) of the forearm *L*_forearm,cog_ is at 42% of arm segment length as measured from the elbow joint. As the fingers of the hand are closed to hold an additional weight (dumb-bell), the cog was assumed to be at *L*_hand_.

The last missing parameter in the simulation setup of the mechanical system is the damping coefficient *d*. This parameter varies between $<0.1\;\frac{\text{Nm}}{\text{rad/s}}$ up to $15\;\frac{\text{Nm}}{\text{rad/s}}$ [46]. The damping coefficient *d* of the elbow joint in this model is set to $5\;\frac{\text{Nm}}{\text{rad/s}}$. This value was determined by a coarse search.

By using the moment arms $L_{ma}^{\text{bic}|\text{tric}}$ calculated in Eqs (11) and (25) for *θ* = 90°, the maximum forces of the two muscles for the contraction dynamics were calculated as follows:

$$\begin{array}{c} \underset{\text{(elbow}\;\text{flexion)}}{\underset{︸}{F_{\text{max}}^{\text{bic}}=\frac{T_{\text{max}}^{\text{flex}}}{L_{ma}^{\text{bic}}\left(90^{\circ }\right)}}},\;\underset{\text{(elbow}\;\text{extension)}}{\underset{︸}{F_{\text{max}}^{\text{tric}}=\frac{T_{\text{max}}^{\text{ext}}}{L_{ma}^{\text{tric}}}}}. \end{array}$$ (28)

$T_{\text{max}}^{\text{flex}|\text{ext}}$ is the maximum torque as calculated in Eq (1).

At this point, all but five parameters of the overall model were set. Only these five free parameters were used in the optimization process. To calculate the angular acceleration of the elbow joint based on the described masses and their distribution, torques which result from the flexor (*biceps* long head + *biceps* short head) and extensor (*triceps* lateral head + *triceps* long head) muscles were summed up. The elbow joint was assumed to be a simple revolute joint (see [37, 41]). Elbow joint angle (*θ*) and angular velocity (*ω*) were computed by numeric integration. Initial values were calculated based on measured elbow angle (*θ*_meas_). Initial angular acceleration (${\dot{\omega }}_{0}$) was determined using the measured angular velocity (*ω*_meas_). Since the shoulder movement is not in the focus of this investigation—the shoulder joint in the simulation was oriented according to the shoulder angles measured in the IMU units which are integrated in the *biceps* sEMG sensors. As described in the experimental paradigm, the subject holds an additional weight *m*_add_ in the hand. This is in addition to the weight of the forearm and the hand. The simulation was conducted in Matlab 2019a using the Simulink and Simscape toolboxes (The Mathworks Inc., Natick,MA, USA). The simulation outputs the course of the elbow joint angle *θ*_sim_ as generated by the model. During the optimization process of the five free parameters, *θ*_sim_ was compared to the measured angle *θ*_meas_.

#### Signal preparation and model optimization

In the optimization process, the goal was to find those values of the remaining five free parameters that minimize the error between simulated elbow angle *θ*_sim_ and measured elbow angle *θ*_meas_. Only the four gain values *k* for the raw sEMG signals of the *biceps* and *triceps* muscle heads (see paragraph on sEMG preprocessing and Fig 4, left side) and the offset $L_{\text{offset}}^{\text{tric}}$ of the musculoskeletal mechanics (see Eq (26)) were not set to subject dependent values and had to be optimized. To get a robust optimization, the start values were estimated before the optimization. The measured signals of the conducted experiments were split into separate intervals according to the respective phase of the movement. At the beginning of each experiment, there was a nearly motionless time interval which is called static phase in the following. This was followed by the first period of the cyclic movements which started at the initial value of *θ*_meas_ = 90°, contained one upwards and one downwards movement of the forearm and ended at the initial value of *θ*_meas_ = 90°. The first three full periods of each experiment were used for the optimization.

Before the start of the optimization, the upper and lower limits of the free parameters had to be estimated to constrain the optimization algorithm during its search. During the static phase, the values of the gains $k_{\text{est}}^{\text{bic}|\text{tric}}$ were estimated. In the static phase, the elbow torque *T*_static_ as generated by the muscles was mainly caused by the arm weight *m*_arm_, the hand weight *m*_hand_ and the additional weight *m*_add_ due to the dumbbell. The estimates of the gains $k_{\text{est}}^{\text{bic}|\text{tric}}$ were calculated based on Eqs (29) and (30).

$$\begin{array}{c} T_{\text{static}}^{\text{bic}|\text{tric}}\left(\alpha \approx 0^{\circ }|180^{\circ }\right)=\text{cos}\left(\alpha \right)\cdot (m_{\text{forearm}}\cdot L_{\text{forearm,cog}}\\ +m_{\text{hand}}\cdot L_{ec\_m,\text{palm}}+m_{\text{add}}\cdot L_{ec\_m,\text{palm}}) \end{array}$$ (29)

$$\begin{array}{c} k_{\text{est}}^{\text{bic}|\text{tric}}=\frac{1}{{\bar{a}}_{\text{static}}^{\text{bic}|\text{tric}}}\cdot \frac{T_{\text{static}}}{T_{\text{max}}} \end{array}$$ (30)

In Eq (29), the angle *α* indicates the posture of the upper arm (*α* ≈ 0° → upper arm pointing downwards, *α* ≈ 180°→ upper arm pointing upwards, measured by the IMU in the EMG-sensors). In Eq (30), ${\bar{a}}_{\text{static}}^{\text{bic}|\text{tric}}$ is the mean muscle activation during the static phase. The estimated gain values $k_{\text{est}}^{\text{bic}|\text{tric}}$ can only be determined in the *lower posture* (*α* ≈ 0°) for the *biceps* muscle and in the *upper posture* (*α* ≈ 180°) for the *triceps* muscle. Based on the estimated value $k_{\text{est}}^{\text{bic}|\text{tric}}$, upper and lower limits of the optimization process for the gains $k_{\text{opt}}^{\text{bic}|\text{tric}}$ were set according to

$$\begin{array}{c} 0.1\cdot k_{\text{est}}^{\text{bic}|\text{tric}}\leq k_{\text{opt}}^{\text{bic}|\text{tric}}\leq 5\cdot k_{\text{est}}^{\text{bic}|\text{tric}} \end{array}$$ (31)

to enforce realistic values. The limits of the offset $L_{\text{offset}}^{\text{tric}}$ were set to ±0.05m.

The five free parameters (parameter set) were optimized in two steps. In the first step, a random walk search was used to scan the overall parameter space in the previously defined limits. This helped to escape a local minimum that was otherwise found for small parameter values when the forearm is mainly driven by gravity. For the random walk, 1044 parameter sets were randomly taken from the interval between zero and one of a uniform distribution (rand(n,m) function, Matlab V2019b, The Mathworks Inc., Natick, MA, USA). Each parameter of the parameter sets was mapped to the interval between the lower and upper limits. For each parameter set, the model was simulated and the sum of squared errors for the elbow angle trajectories was stored. Afterwards, the parameter set with the smallest squared error was used in the next step. In the second optimization step, a gradient descent algorithm (lsqnonlin, Matlab V2019b) was initialized based on the parameter set with the lowest error from the first step. The lower limits of gain values ($k_{\text{opt}}^{\text{bic}|\text{tric}}$) (Eq (31)) were set to zero in this step to allow the optimizer to fade out an EMG. A maximum of 20 iterations and a minimum step size of 1 ⋅ 10^−3^ were chosen as termination criteria. Trust-region-reflective was used as optimization algorithm. For each of the subjects, the eight experiments were optimized by tuning the five free parameters while the previously fixed 37 parameters remain unchanged throughout all experiments.

### 2.3 Characteristics of chosen error and quality scores

To evaluate the predictive performance of the musculoskeletal model, EMG-data as recorded in the experiments (see section 2.1) was fed into the model and the model was used to simulate the course of the elbow angle *θ*_sim_. Based on the difference between simulated course *θ*_sim_ and real course *θ*_meas_ of the elbow angle the free parameters of the model were optimized and evaluated. For the comparison between measured angle and simulated angle in section 3, the mean absolute error (*MAE*) was calculated as shown in Eq (32).

$$\begin{array}{c} MAE\left(\theta_{\text{sim}}\right)=\frac{\sum_{i=1}^{n}\theta_{\text{sim},i}-\theta_{\text{meas},i}}{n} \end{array}$$ (32)

With *n* being the number of measured values during one experiment. The mean absolute error (*MAE*) is an error measure which is independent of the length *n* of the measurements and allows comparison of results across experiments with different recording times. In addition, the normalization of the *MAE* to the range of the *θ*_meas_ as shown in Eq (33) allows for the comparison across different experiments/subjects with different movement behavior.

$$\begin{array}{c} nMAE\left(\theta \right)=\frac{MAE(\theta )}{\text{range(}\theta_{\text{meas}})}\;\text{with}\;\text{range}\left(\theta_{\text{meas}}\right)=\text{max}\left(\theta_{\text{meas}}\right)-\text{min}\left(\theta_{\text{meas}}\right) \end{array}$$ (33)

**Fig 6:**
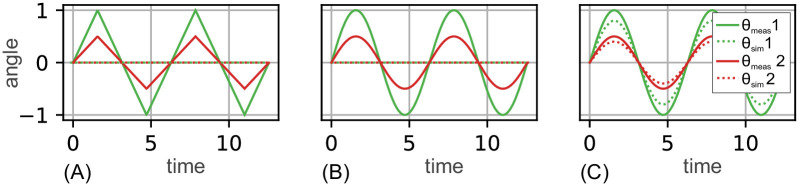
Comparison between *MAE* and *nMAE*. In each subfigure (A), (B) and (C) there are two exemplary elbow joint movements of different waveform and amplitude. Waveforms in solid green lines have an amplitude of 1, those in solid red line of 0.5. In (A) and (B) the simulated prediction *θ*_sim_ (dashed lines in green|red) is constantly at zero. C) gives an alternative example for a simulated *θ*_sim_ at 80% of the signal amplitude. The resulting error is independent of the range, but dependent on the signal form as shown in Table 2.

The behavior of the two error measures *nMAE* and *MAE* for different signal shapes and amplitudes is illustrated by the imaginary movement data *θ* for an elbow joint in Fig 6. In Fig 6(A) two triangular curve shapes with different amplitude (${\hat{\theta }}_{\text{meas}}=0.5|1.0$ as solid line in red|green) are shown. In this example, it was assumed that the simulation model for both cases makes a constantly incorrect prediction of *θ*_sim_ = 0 (dashed line in red|green) which would be the mean posture of the given imaginary elbow movement. In Fig 6(B) two sinusoidal curve shapes again with different amplitude (${\hat{\theta }}_{\text{meas}}=0.5|1.0$ as solid line in red|green) are shown together with the again incorrect simulation model prediction of *θ*_sim_ = 0 (dashed line in red|green).

The respective *MAE* and *nMAE* are given in the first two rows of Table 2 for Fig 6(A) and in row three and four for Fig 6(B). It can be seen that the *nMAE* is independent of the signal amplitude (green vs. red) but sensitive to signal shape ((A) vs. (b)). Fig 6(C) exemplarily shows simulation model predictions (dashed lines in red|green) that are at 80% of the amplitude of the respective measurements (solid lines in red|green) again resulting in the same *nMAE* value. An *nMAE* of 0.1 at a maximum angle range of 90° corresponds to a mean deviation of 0.1 ⋅ 90° = 9° between *θ*_meas_ and *θ*_sim_. The minimal value of the *nMAE* is zero.

**Table 2:** Values of *MAE*, *nMAE* and *QS* for different curve shapes and amplitudes as given in Fig 6.

subfigure, shape	curve	${\hat{\theta }}_{\text{meas}}$	${\hat{\theta }}_{\text{sim}}$	MAE	nMAE	QS
(A) triangular	green	1	0	0.50	0.25	0
(A) triangular	red	0.5	0	0.25	0.25	0
(B) sinusoidal	green	1	0	0.63	0.32	0
(B) sinusoidal	red	0.5	0	0.32	0.32	0
(C) sinusoidal	green	1	1*0.8	0.13	0.06	0.8
(C) sinusoidal	red	0.5	0.5*0.8	0.06	0.06	0.8

Note that the *nMAE* is not independent of the waveform. Therefore, it is only conditionally suitable for the comparison between different movements of subjects.

To improve the comparability between subjects, a quality score *QS* was introduced which normalizes the *nMAE* by using the *nMAE* of *θ*_const_ (a constant mean elbow joint angle) per subject and experiment. This results in a ratio that should range between slightly below zero and one. This value is calculated as shown in Eq (34).

$$\begin{array}{c} QS=1-\frac{nMAE(\theta_{\text{sim}})}{nMAE(\theta_{\text{const}})} \end{array}$$ (34)

The quality score *QS* is zero if *θ*_sim_ is equal to *θ*_const_, i.e. if no or only a very small movement is apparent. This is the case in the examples in Fig 6(A) and (6B) for which the QS values are shown in the first four rows of Table 2. The quality score can become smaller than zero if *θ*_sim_ is not equal to *θ*_const_ and has a constant curve. A quality score of greater than zero means a simulation in which *θ*_sim_ is not constant, i.e. the forearm is moving. In the model described in section 2.2, this movement can only occur through an active force due to the sEMG signals. With a quality score of one, *θ*_sim_ corresponds to the course of *θ*_meas_. Since the quality score is normalized to the respective *nMAE* at *θ*_const_, the quality score is independent of the signal form.

## 3 Results

**Fig 7:**
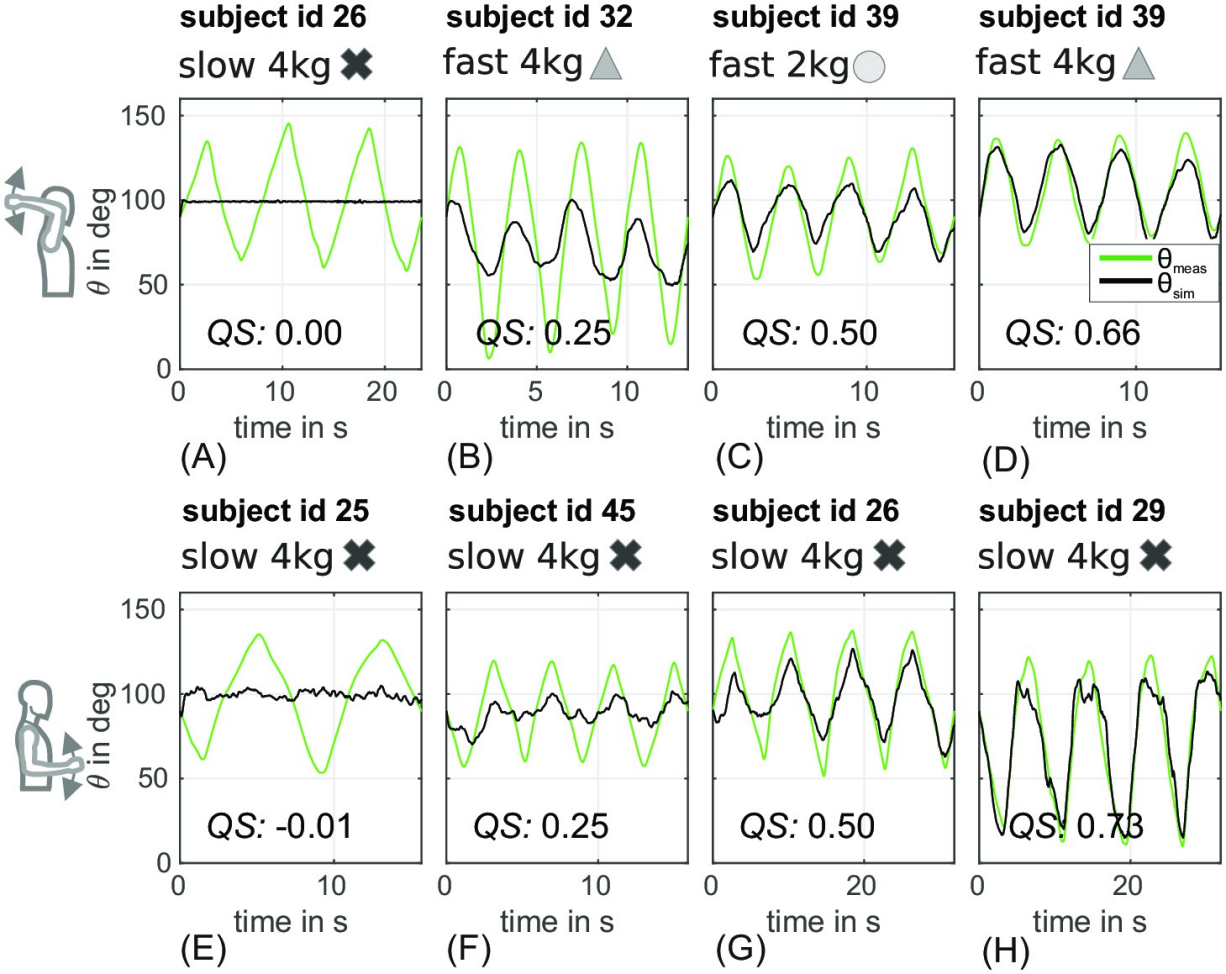
Selected examples of forearm movements (elbow angle *θ*_meas_) with quality scores *QS* close to [0, 0.25, 0.5, 0.75]. Upper row (A-D) shows four results for the *upper posture*, the lower row (E-H) for the *lower posture*. The first column (A) and (E) show two results with *QS* close to 0; (B) and (F) a *QS* close to 0.25; (C) and (G) a *QS* close to 0.5 and (D) and (H) a *QS* close to 0.75. Different experiments were marked with symbols (cross, square, triangle, circle).

Subjects performed forearm movements according to the experimental paradigm as described in section 2.1 (2 upper arm postures, 2 weights (dumbbell, [2kg, 4kg]), 2 movement speeds ([0.25Hz (slow) and 0.5Hz (fast)]) for the forearm movement). A metronome was used as acoustic reference. The subjects were asked to move the forearm periodically in a continuous manner reaching the reversal points of the movement in time with the rhythmic acoustic signal. This requirement was expected to lead to a rather sinusoidal movement behaviour of the elbow angle. Fig 7 shows selected examples for different courses of *θ*_meas_ (green) for the *upper* (A-D) and *lower posture* (E-H). It can be seen that subjects sometimes tended to accelerate strongly at the extreme positions leading to a more triangular rather than sinusoidal waveform.

**Fig 8:**
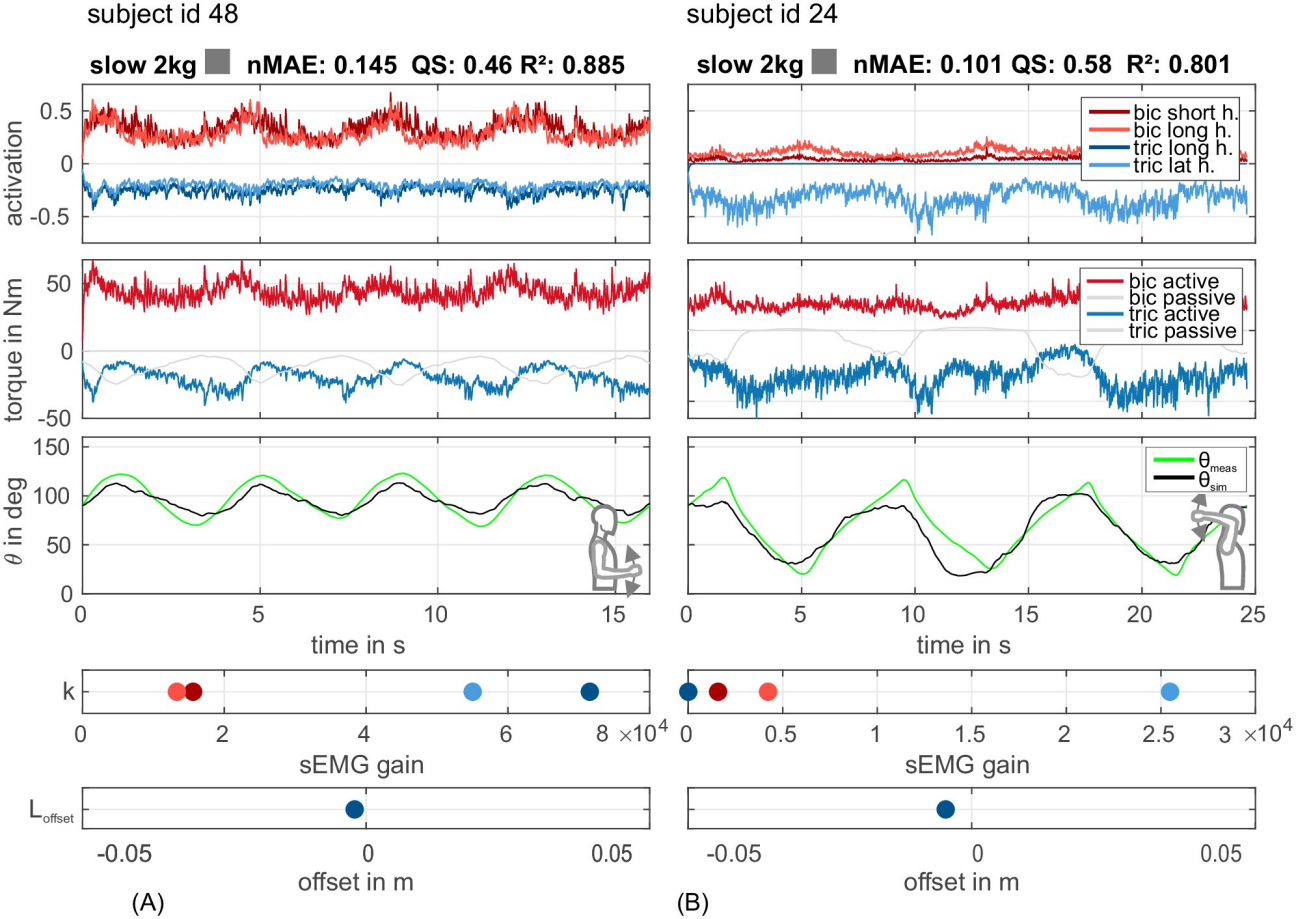
Optimization results for two exemplary subjects in the same experimental condition (slow, 2 kg) but at different postures. (A) *Lower posture* with the movement course having a more sinusoidal character (subj. 48). (B) *Upper posture* with movement course having a more triangular shape (subj. 24). First row shows neural activation of both *biceps* muscle heads in shades of red and two *triceps* muscle heads in shades of blue (*triceps* data is shown mirrored). Second row shows elbow joint torque components according to the contributing muscles. As shown in Fig 4, both muscle heads are combined in one contraction dynamics (one active torque for each muscle group). Gray signals are torque components originating from passive muscle forces. Third row shows measured (*θ*_meas_, green) and simulated (*θ*_sim_, black) elbow joint angle course. Below the time curves of the free parameters are shown (colour code according to the respective muscles).

As described, 36 parameters of the simulation model were set based on measurements of anatomical features and values from literature and were therefore valid for a subject independently of the experimental conditions. The remaining 5 free parameters were optimized per experimental condition. Fig 7 depicts the simulation results *θ*_sim_ in black. The subjects and experiments were chosen exemplary to give an impression of the quality score *QS* in approximately 25% steps. Fig 8 course shows data from two subjects in the same experimental condition (slow, 2 kg) but at different postures ((A) *lower posture*, (B) *upper posture*). The subject in Fig 8(A) shows a more sinusoidal course of *θ*_meas_ as compared to the more triangular course in Fig 8(B).

**Fig 9:**
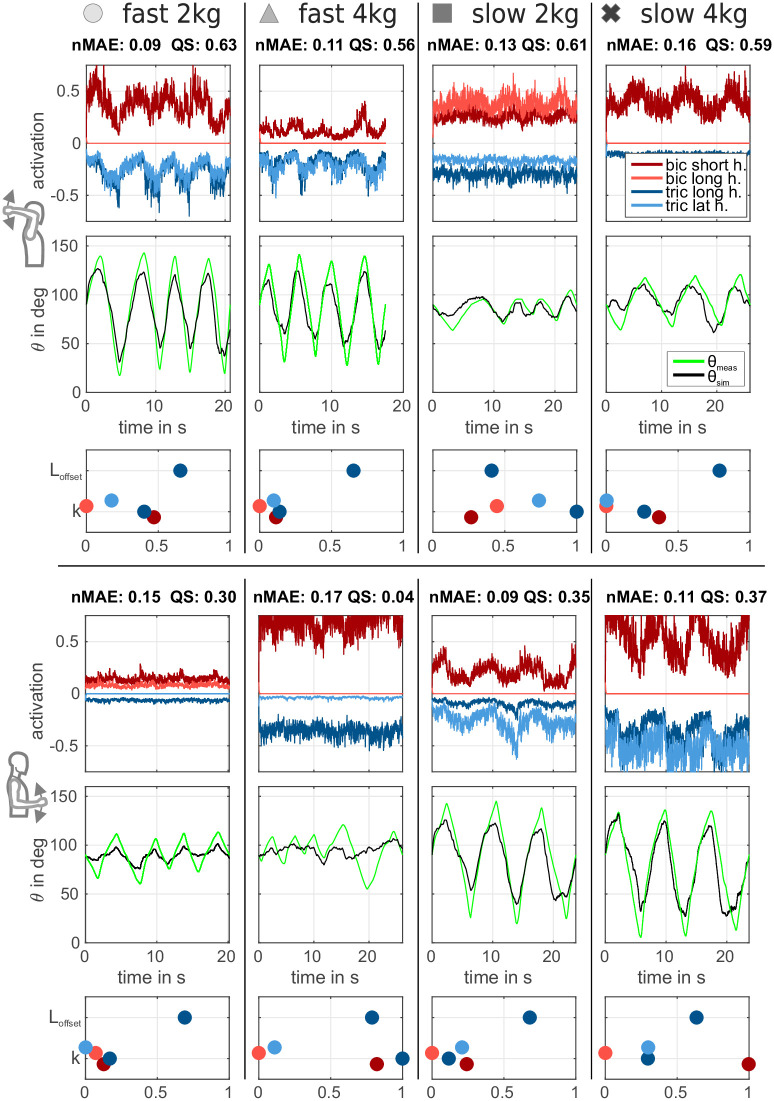
Comparison of all experiments for one subject (subj. id 21). The selected subject has achieved the highest mean of the *QS* across all experiments combined. The top part shows the experiments in the *upper posture*, the bottom part those in the *lower posture*. For each experiment, curves are depicted with the same color coding as in Fig 8 (torque curves are not shown). Columns represent different experiments which were marked with symbols (cross, square, triangle, circle).

The fourth and fifth rows show the values of the optimised 5 free parameters. In particular, the values for the sEMG amplification factors *k* vary with the EMG signal strength/quality (location of the applied electrodes and quality of the electrode-skin interface). A *k* of 0 means that this EMG was faded out by the optimiser. As described, 36 model parameters were fixed for one subject and used across all experiments. Among the subjects these parameters differ but were not optimized. The 5 free model parameters per subject are used for the adaptation to postures and experimental conditions. A comparison of the four experiments per posture for one subject is shown in Fig 9. The values for the *k* and *L*_offset_ are given in a normalized form. The boundaries for the optimization process (see section 2.2) are used for the normalization.

**Fig 10:**
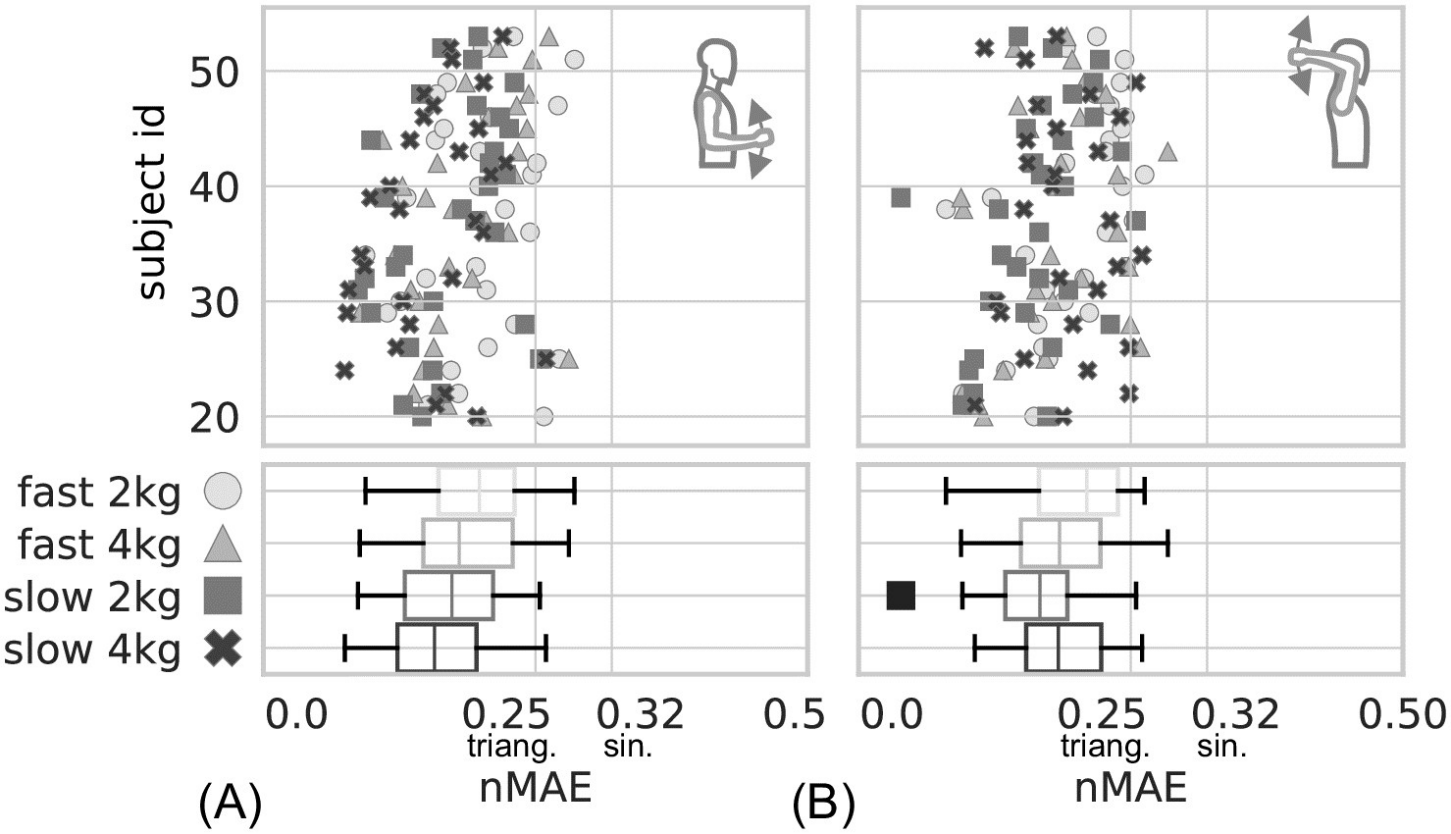
The *nMAE* for all subjects in the two different postures. In (A), results for the *lower posture*, and in (B) results for the *upper posture* are shown. Vertical gray lines at 0.25 (triangular) and 0.32 (sinusoidal) indicate the theoretical *nMAE* for a constant prediction *θ*_const_ for the respective signal as a reference. The experiments with different movement speeds and different additional weights are shown in four different shades of gray and with different symbols (cross, square, triangle, circle). The diagrams at the top show individual results. The bottom diagrams show aggregated results in individual box and whisker plots for speed and weight combinations. The chronological sequence of the experiments is from top to bottom. Data was published in [23].

In total, experiments were conducted with 31 subjects (Data was published in [23]) and the simulation models were parameterized (36 parameter for each subject) and optimized (5 free parameters per experimental condition). The quality of the sEMG-based model predictions for the movement trajectories *θ*_sim_ of the forearm with respect to the measured trajectories *θ*_meas_ is depicted in Fig 10 as *nMAE* for all subjects. Fig 10(A) contains the results for the *lower posture* (*biceps* dominant), (B) for the *upper posture* (*triceps* dominant).

In the upper panel of Fig 10(A), the *nMAE* values for each subject are plotted in one row. The *nMAE* result for each experimental conditions is marked with a symbol (cross, square, triangle, circle) and thus represents the optimization of the five free parameters for the respective condition. The lower panel of Fig 10(A) shows summarized data of the upper panel as box plots, one for each experimental condition (speed-weight-combination). Values for the respective quartiles are given in the top part of S5 Table. The median values (Q2) of the *nMAE* for the *lower posture* correspond—according to the arrangement in the lower panel of Fig 10(A)—from top to bottom (fast-2 kg ≙ 0.20, fast-4 kg ≙ 0.18, slow-2 kg ≙ 0.17, slow-4 kg ≙ 0.16).

The upper panel of Fig 10(B) follows the same structure as the upper panel in Fig 10(A) but for the *upper posture*. The lower panel of Fig 10(B) also shows summarized data as box plots. The median values (Q2) of the *nMAE* for the *upper posture* correspond—according to the arrangement in the lower panel of Fig 10(B)—from top to bottom (fast-2 kg ≙ 0.21, fast-4 kg ≙ 0.19, slow-2 kg ≙ 0.17, slow-4 kg ≙ 0.18).

**Fig 11:**
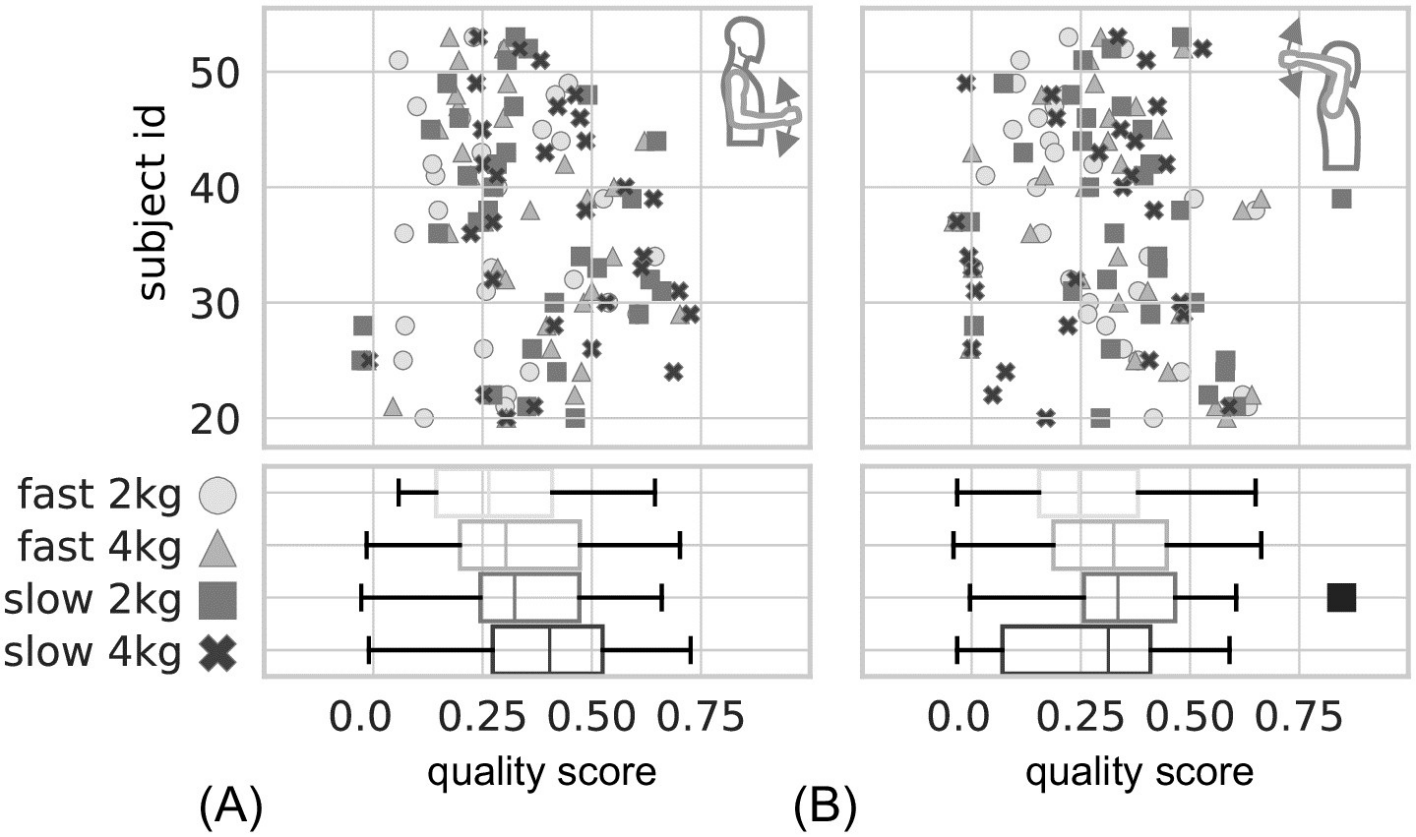
Quality score *QS* for all subjects in two different postures. In (A), results for the *lower posture*, and in (B) results for the *upper posture* are shown. The general structure of the graphs is the same as in Fig 10. The quality score is calculated as shown in Eq (34).

As supplement to Fig 10, the quality of the sEMG-based model predictions for the movement trajectories is depicted in Fig 11(A) and 11(B) as quality score *QS* for all subjects also represented as box plots in the respective lower panels.

The values for the respective quartiles of the *QS* box plots are given in S6 Table. The median values (Q2) of the *QS* for the *lower posture* are (fast-2 kg ≙ 0.26, fast-4 kg ≙ 0.30, slow-2 kg ≙ 0.32, slow-4 kg ≙ 0.40) for the *lower posture* and (fast-2 kg ≙ 0.25, fast-4 kg ≙ 0.33, slow-2 kg ≙ 0.34, slow-4 kg ≙ 0.31) for the *upper posture*. The prediction qualities for both postures show medians of the *QS* above 0.25 which corresponds to a fit of the simulated data to the measured data which is visibly similar.

## 4 Discussion

In this work, a model has been presented that predicts limb movements of the human forearm based on sEMG signals from the main muscles involved in the biological actuation of the elbow joint. The prediction is based on a biomechanical model of the humerus, forearm and elbow joint including the associated muscles. Flexion of the elbow mainly relies on three muscles, the two-headed *biceps brachii*, the single-headed *brachioradialis* and the single-headed *brachialis*. The latter is located below the *biceps*. Some weaker flexor muscles located in the forearm were omitted. Elbow extension is also based on two muscles, the three-headed *triceps brachii* and the single-headed *anconeus* (the latter also tightens the joint capsule). Although in principle all these muscles are involved in the joint movement of the elbow, only the sEMGs of those muscles—respectively muscle heads—were recorded that can be easily measured from the outside (*lateral* and *long head* of *triceps* and *short* and *long head* of *biceps*). This was done on the assumption that this restriction is important for applications in exoskeletal controls which require a simple electrode placement that is as unaffected as possible by positional shifts [20, 21]. At the same time, this reduced sEMG electrode setup might be associated with a reduction in the quality of movement prediction (see quality score). Biomechanical prediction models use a variety of parameters such as muscle lengths, locations and orientation of points of the skeletal subsystem under consideration, or physiological parameters such as time constants of muscle activation dynamics, to name a few. For the prediction model in this study, the total number of parameters that can be set in principle is 42 (see section 2.2). On the one hand, setting such a number of parameters individually, e.g. for modelling motion prediction within exoskeletal controls, is not practical. For an online optimization of all parameters, on the other hand, plausible physiological constraints would have to be met. The strategy chosen here is to measure easily accessible and palpable body points of the shoulder (*acromion*), elbow (*medial/lateral epicondyles* and *olecranon*) and wrist (*styloid processes* of *radius/ulna*) to estimate desired internal, not directly observable lengths via known correlations (respective references are given in section 2.2). Together with a maximum force measurement of the forearm, the number of parameters still to be set could be reduced from 42 to 5 (see section 2.2).

Assuming that these 5 parameters are sufficient to individualise the prediction model, movement experiments were carried out with 31 subjects in which cyclic forearm movements were each measured at two different speeds and two different weights in two different postures. The data set in this study is therefore quite extensive and uses more subjects than comparable work which fits movement models based on a smaller number of subjects and conditions (e.g. 1 to 5 subjects in [32, 47–50]). The data set of this study has been made available in a separate publication [23] for further scientific use. The corresponding results of the described modelling task were presented in detail in section 3. Two aspects in particular should be emphasised. Firstly, the results show individualised per-subject models in which 37 out of 42 parameters were determined by a single adjustment based on anatomical measurements, i.e. independent of experimental conditions. The downstream optimization of 5 parameters in relation to the concrete experimental conditions (posture, weight, speed) can therefore be carried out more quickly and can be used in later work as a basis for online adaptations, e.g. during the ongoing operation of an exoskeleton. Secondly, the quality of the prediction or fit of the forearm movement was expressed in different error or quality measures. In the representation as quality score (QS), the median of the QS varies over all experimental conditions and over all subjects between approximately 0.25 and about 0.4. Visual inspection of the results suggests that the median and the interquartile range (*IQR*) of the *nMAE* for experiments with 4 kg additional weight are lower than for the experiments with 2 kg additional weight. Also, the median and *IQR* of the *nMAE* for experiments with slow movements are lower than for those with fast movements. The median and the *IQR* of the *nMAE* for experiments with 4 kg are almost equal for both speeds. However, the slow 4 kg experiments show higher *nMAE* values than in the same experiments in the *lower posture*. This might be related to a stronger/faster fatigue of the subjects during movements with the heavier weight in the unfamiliar *upper posture*. The depiction as *QS* values shows the same tendencies as already described for the *nMAE* values in Fig 10.

Fig 7 shows exemplarily to which movement curves these QS values lead to. In particular, the phase (correct direction of the respective motion) of the low-frequency components of the movement curves seem to be mainly correct. Deviations between the actual and predicted course of the forearm movements seem to be more strongly reflected in the amplitude differences of the movements than in the phase of the movement. This is most likely explained by the fact that four of the freely adjustable parameters are the gain factors *k* of the *sEMG* signals and therefore influence the active torque/active movement.

Future work includes the further development of the domain model shown here towards a hybrid model that integrates additional, data-driven (ML-based) sub-models. Such a hybrid approach could, on the one hand, further increase the prediction quality and, on the other hand, identify and address over-simplifications in sub-models of the existing domain model. Such a hybrid approach should also combine the explainability of a pure domain model with the adaptability of a purely data-driven (black box) approach [51]. The quality of the movement prediction of the domain model in this work will serve as a reference for the described next steps.

## Supporting information

S1 Table Parameters for sEMG preprocessing. (PDF)

S2 Table Parameters for activation dynamics submodel. (PDF)

S3 Table Parameters for contraction dynamics submodel. (PDF)

S4 Table Parameters for musculoskeletal submodel. (PDF)

S5 Table Values of quartiles for *nMAE* in different postures as shown in Fig 10. (PDF)

S6 Table Values of quartiles for quality score *QS* in different postures as shown in Fig 11. (PDF)
