# Supplementary material for: sEMG-based prediction of human forearm movements utilizing a biomechanical model based on individual anatomical/ physiological measures and a reduced set of optimization parameters
Source: PLoS One. 2023 Aug 3;18(8):e0289549. doi: 10.1371/journal.pone.0289549 (PMC10399825; doi:10.1371/journal.pone.0289549)
Supplement: S1 Table — (PDF) [file pone.0289549.s001.pdf]

S1 Table. Parameters for sEMG preprocessing.

name	value	source
k	subject dependent	optimization
f_{low}, f_{high}	4 Hz, 400 Hz	[27]
