# Supplementary material for: sEMG-based prediction of human forearm movements utilizing a biomechanical model based on individual anatomical/ physiological measures and a reduced set of optimization parameters
Source: PLoS One. 2023 Aug 3;18(8):e0289549. doi: 10.1371/journal.pone.0289549 (PMC10399825; doi:10.1371/journal.pone.0289549)
Supplement: S2 Table — (PDF) [file pone.0289549.s002.pdf]

S2 Table. Parameters for activation dynamics submodel.

name	value	source
τ_{act}	17.3 ms	step response equal to [28]
β	0.35	step response equal to [28]
A	-0.25	coarse search
