# Supplementary material for: sEMG-based prediction of human forearm movements utilizing a biomechanical model based on individual anatomical/ physiological measures and a reduced set of optimization parameters
Source: PLoS One. 2023 Aug 3;18(8):e0289549. doi: 10.1371/journal.pone.0289549 (PMC10399825; doi:10.1371/journal.pone.0289549)
Supplement: S3 Table — (PDF) [file pone.0289549.s003.pdf]

S3 Table. Parameters for contraction dynamics submodel.

name	value	source
K_v	5	[29–31]
N	1.4	[17]
c	$ln(0.05)$	[29]
w	0.5	[7, 8, 16, 30]
K_p	2.78	[17]
$v_{\max}$	$10\,s^{-1} \cdot L_{oM}$	[17, 32]
