# Supplementary material for: sEMG-based prediction of human forearm movements utilizing a biomechanical model based on individual anatomical/ physiological measures and a reduced set of optimization parameters
Source: PLoS One. 2023 Aug 3;18(8):e0289549. doi: 10.1371/journal.pone.0289549 (PMC10399825; doi:10.1371/journal.pone.0289549)
Supplement: S4 Table — (PDF) [file pone.0289549.s004.pdf]

S4 Table. Parameters for musculoskeletal submodel.

name	value	source
B^{bic}	0.0472 m	[38]
$\bar{r}^{\mathrm{bic}}_{L_{ac,ec\_l},A}$	0.9	derived from [39] and [40]
$\bar{r}^{\mathrm{bic}}_{LMTC0,A}$	1.07	derived from [41]
$\bar{r}^{\mathrm{bic}}_{LMTC0,LM0}$	2.48	derived from [41]
$\bar{r}^{\mathrm{tric}}_{LMTC0,LM0}$	3.5	derived from [41]
male: $\bar{L}_{ac,ax}$	0.121 m	[42, p. 346]
female: $\bar{L}_{ac,ax}$	0.099 m	[42, p. 346]
male: $\bar{r}^{\mathrm{tric}}_{Lac,ec\_l,A}$	1.22	derived from [42, p. 346, 348] (based on [43])
female: $\bar{r}^{\mathrm{tric}}_{Lac,ec\_l,A}$	1.20	derived from [42, p. 346, 348] (based on [43])
$L^{\mathrm{bic}}_{M,0}$	subject dependent	eq. (16)
$L^{\mathrm{tric}}_{M,0}$	subject dependent	eq. (24)
$L^{\mathrm{tric}}_{\mathrm{ma}}$	subject dependent	eq. (25)
$L^{\mathrm{tric}}_{\mathrm{offset}}$	subject dependent	optimization
