# Supplementary material for: sEMG-based prediction of human forearm movements utilizing a biomechanical model based on individual anatomical/ physiological measures and a reduced set of optimization parameters
Source: PLoS One. 2023 Aug 3;18(8):e0289549. doi: 10.1371/journal.pone.0289549 (PMC10399825; doi:10.1371/journal.pone.0289549)
Supplement: S5 Table — (PDF) [file pone.0289549.s005.pdf]

S5 Table. Values of quartiles for $nMAE$ in different postures as shown in fig. 10.

**lower posture**						
**weight in kg**	**speed**	Q0	Q1	Q2 (median)	Q3	Q4
2	fast	0.094	0.161	0.198	0.231	0.286
4	fast	0.089	0.147	0.180	0.229	0.281
2	slow	0.087	0.130	0.173	0.211	0.254
4	slow	0.075	0.123	0.157	0.196	0.260
**upper posture**						
**weight in kg**	**speed**	Q0	Q1	Q2 (median)	Q3	Q4
2	fast	0.080	0.166	0.210	0.238	0.263
4	fast	0.094	0.149	0.185	0.222	0.284
2	slow	0.095	0.135	0.166	0.192	0.255
4	slow	0.107	0.154	0.183	0.223	0.260
