# Supplementary material for: sEMG-based prediction of human forearm movements utilizing a biomechanical model based on individual anatomical/ physiological measures and a reduced set of optimization parameters
Source: PLoS One. 2023 Aug 3;18(8):e0289549. doi: 10.1371/journal.pone.0289549 (PMC10399825; doi:10.1371/journal.pone.0289549)
Supplement: S6 Table — (PDF) [file pone.0289549.s006.pdf]

S6 Table. Values of quartiles for quality score *QS* in different postures as shown in fig. 11.

**lower posture**						
**weight in kg**	**speed**	Q0	Q1	Q2 (median)	Q3	Q4
2	fast	0.058	0.144	0.264	0.409	0.645
4	fast	-0.015	0.198	0.303	0.473	0.702
2	slow	-0.028	0.245	0.323	0.471	0.660
4	slow	-0.010	0.273	0.404	0.525	0.726
**upper posture**						
**weight in kg**	**speed**	Q0	Q1	Q2 (median)	Q3	Q4
2	fast	-0.033	0.154	0.245	0.381	0.650
4	fast	-0.042	0.187	0.325	0.447	0.663
2	slow	-0.004	0.257	0.335	0.466	0.606
4	slow	-0.033	0.070	0.313	0.409	0.590
